# Ginsenoside Rb1 regulates CPT1A deacetylation to inhibit intramuscular fat infiltration after rotator cuff tear

**DOI:** 10.1016/j.isci.2024.110331

**Published:** 2024-06-20

**Authors:** Yuesong Yin, Zili Wang, Yian Yang, Minren Shen, Hai Hu, Chuanshun Chen, Hecheng Zhou, Zheng Li, Song Wu

**Affiliations:** 1 Department of Orthopaedics, The Third Xiangya Hospital, Central South University, No. 138 Tongzipo Road, Changsha 410013, China; 2 Department of Oncology, The Third Xiangya Hospital, Central South University, No. 138 Tongzipo Road, Changsha 410013, China; 3 NHC Key Laboratory of Carcinogenesis, Cancer Research Institute, School of Basic Medical Sciences, Central South University, Changsha, China

**Keywords:** Musculoskeletal anatomy, Cell biology

## Abstract

Fat infiltration (FI) in the rotator cuff muscle is associated with poor clinical outcomes and failed repair of rotator cuff tears (RCTs) in patients. In this study, we aimed to investigate the function of ginsenoside Rb1 in inhibiting FI in muscles after RCT and its underlying molecular mechanism. After TT modeling, mice treated with Rb1 for 6 weeks showed lower FI in the SS muscle compared with mice in the control groups and those treated with other ginsenoside components. Mechanically, Rb1 binds to the NAD+ domain of SIRT1, activating its expression and enzyme activity. This activation stimulates the deacetylation of CPT1A at site K195, thereby promoting fatty acid β-oxidation in adipocyte cells and improving lipolysis. These findings suggest that Rb1 is a potential therapeutic component for improving the outcomes of patients with RCTs.

## Highlights

• We found that ginsenoside Rb1 has a therapeutic effect on rotator cuff muscle FI
• We explore that Rb1 activates the enzymatic activity of Sirt1 and modifies Cpt1a
• We investigate the effects of different deacetylation sites of Cpt1a on its function

## Graphical abstract

**Figure:**
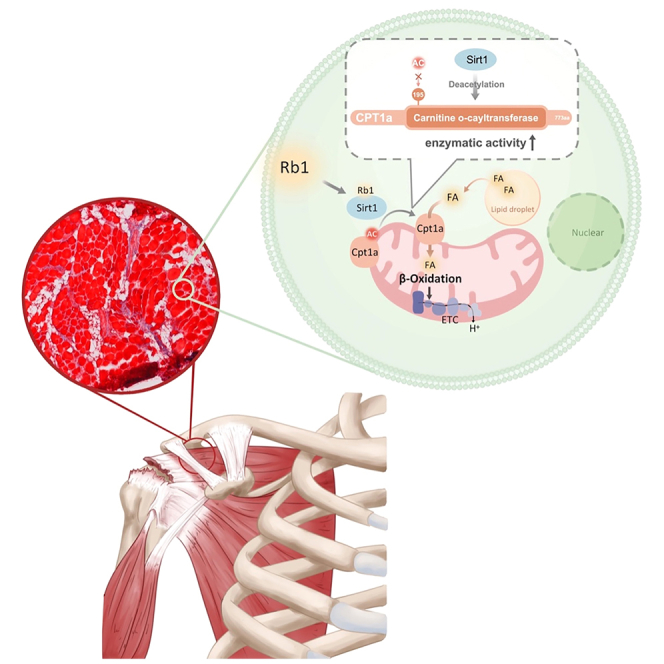

## Introduction

Shoulder pain ranks third among skeletal muscle pain symptoms, and rotator cuff tears (RCTs) are one of the main causes.^1^^,^^2^ Untreated RCT leads to muscle degeneration, including muscle fat infiltration (FI), fibrosis, and muscle atrophy, impairing muscle function. Various clinical studies have found that FI persists in the rotator cuff muscle even after successful repair surgery and is associated with poor functional prognosis and a risk of re-tears.^3^^,^^4^ Intramuscular FI seriously affects shoulder joint function, weakens the rotator cuff muscle, and increases the degree of pain.^5^

Amibegron induces the browning and differentiation of adipose tissue in the supraspinatus muscle (SS), consequently improving rotator cuff degeneration.^6^^,^^7^ Additionally, trichostatin A can reduce FI of the rotator cuff muscles and improve shoulder joint function after massive tendon tears (TTs).^8^ However, no effective treatment for FI in the rotator cuff muscle has been described in clinical guidelines, necessitating the identification of more treatment methods to alleviate shoulder pain symptoms and improve joint function in patients with RCT.

Traditional Chinese medicines represent a valuable source of novel drugs. The use of ginseng (*Panax ginseng* CA MEY) has been reported in the compendium of Materia Medica for more than 2,000 years. Ginsenosides are small molecules that constitute the main functional components of ginseng. Currently, ginsenoside preparations are used worldwide for clinical use and clinical trials for diseases such as non-small cell lung cancer and liver disease.^9^^,^^10^ Ginsenosides promote glucose and lipid metabolism, and ameliorate obesity, metabolic syndrome, and other diseases.^11^ Black ginseng and Rb1 exert anti-obesity effects by inducing browning in 3T3-L1 cells and primary white adipocytes via AMPK-mediated pathway activation.^12^ Ginsenoside Rc downregulates Cebp/α in adipocytes while reducing lipogenesis.^13^ Ginsenoside Rd can treat obesity in mice fed a high-fat diet by promoting the browning of white fat and inducing thermogenesis in a cAMP-dependent manner.^14^ Ginsenoside Rg2 reduces adipogenesis via the AMPK pathway in 3T3-L1 cells and obese mice.^15^

In this study, to detect various ginsenosides potential treatment effect in intramuscular FI, we constructed a C57/BL6 mouse model of TT to simulate intramuscular FI after RCT. AS the results, Ginsenoside Rb1 decreased intramuscular FI most significantly and improves shoulder joint function in TT mice. Mechanistically, Rb1 regulated CPT1A deacetylation via SIRT1 and enhanced fatty acid β-oxidation to improve lipolysis. This study provides direct scientific evidence of the potential therapeutic effects of Rb1 in alleviating intramuscular FI in patients with RCT.

## Results

### Ginsenoside Rb1 inhibits fat infiltration of supraspinatus muscle and improves shoulder joint function in tendon tear mice

**Figure 1:**
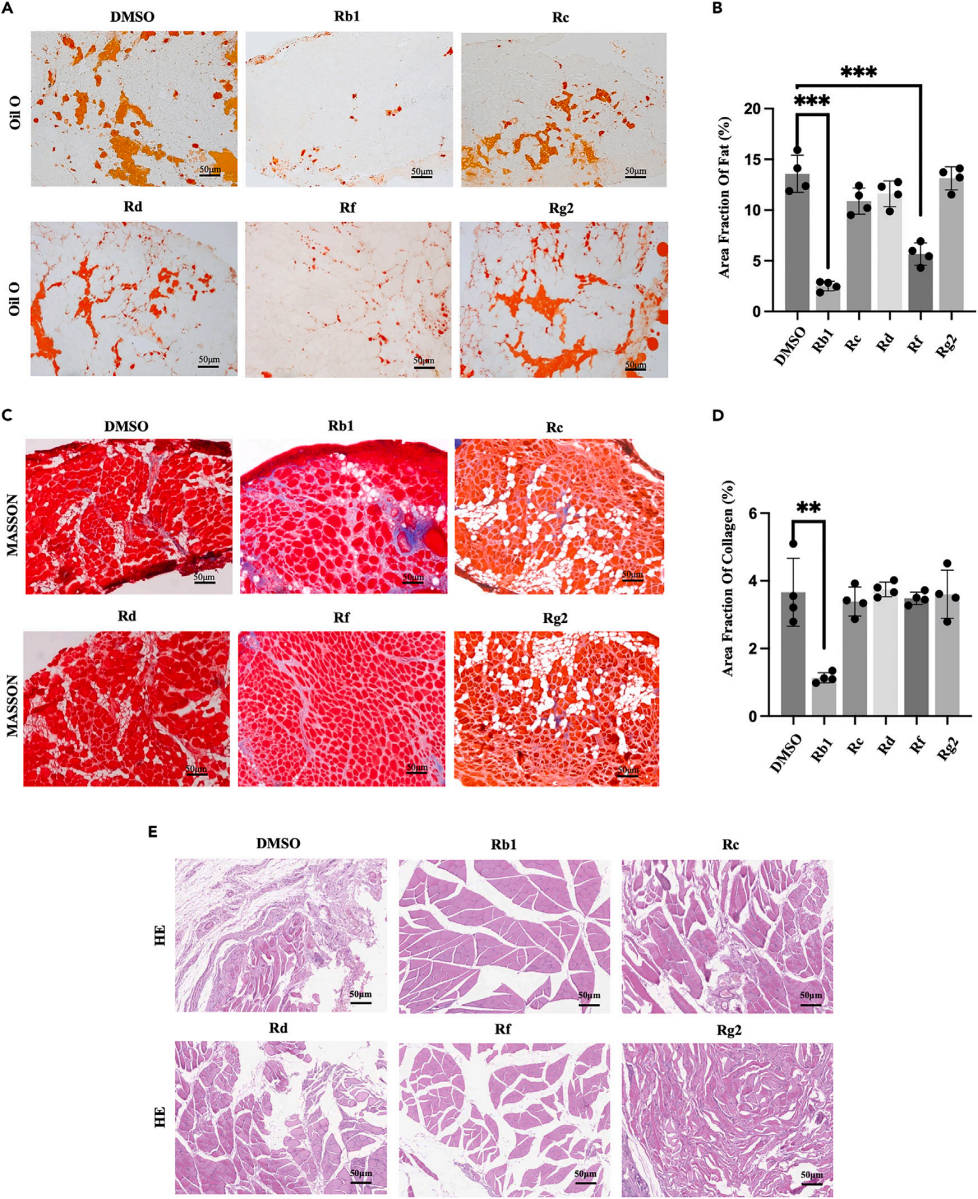
The effect of ginsenosides on fat infiltration in supraspinatus muscle of tendon tear mouse Histological analysis of supraspinatus (SS) muscle in mouse tendon tear (TT) modeling mouse treated with different ginsenoside components or DMSO. (A) Oil Red O staining of the SS muscle in TT mouse treated with DMSO, Rb1, Rc, Rd, Rf, Rg2. Scale bars: 50μm. (B) Fat area fraction analysis of Oil Red O staining. Fat area fraction (%) = area of Oil Red O staining (red)/entire sample area. (C) Masson Trichrome staining of SS muscle in TT mouse treated with DMSO, Rb1, Rc, Rd, Rf, Rg2. Scale bars: 50μm. (D) Collagen area fraction analysis of Masson Trichrome staining. Collagen area fraction (%) = area of collagen staining (blue)/entire sample area. (E) Hematoxylin and eosin (H&E) staining of the SS muscle in TT mouse treated with DMSO, Rb1, Rc, Rd, Rf, and Rg2. Scale bars: 50μm.∗∗*p* < 0.01, ∗∗∗*p* < 0.001.

Initially, we aimed to establish a mouse model of shoulder cuff injury. Among the existing mouse shoulder cuff injury models, the TT model effectively reflects clinical conditions, such as FI and fibrosis in the shoulder cuff muscles.^16^ We also found that FI in the SS muscle appeared at 3 weeks and became more obvious at 6 weeks after surgery, accompanied by reduced upper limb functionality of mice (Figures S2A–S2I). Therefore, we treated mice with an intraperitoneal injection of ginsenoside components from the same day of surgery for 6 weeks and observed. These 5 kinds of ginsenoside components have been reported to affect fat metabolism, including Rb1, Rc, Rd, Rf, and Rg2.^11^^,^^12^^,^^13^^,^^14^^,^^15^^,^^17^ Oil Red O staining revealed that the size and number of lipid droplets in the SS muscle of Rb1 treatment groups were smaller than those in the control and other ginsenoside treatment groups (Figures 1A and 1B). Moreover, Hematoxylin and eosin (H&E) staining and Masson’s trichrome staining revealed that the number of intermuscular fibers in the Rb1 treatment group was lower than that in the control and other groups (Figures 1C–1E). These results show that Rb1 can significantly inhibit SS muscle FI after injury, thus partially improving the atrophic injury caused by FI.

**Figure 2:**
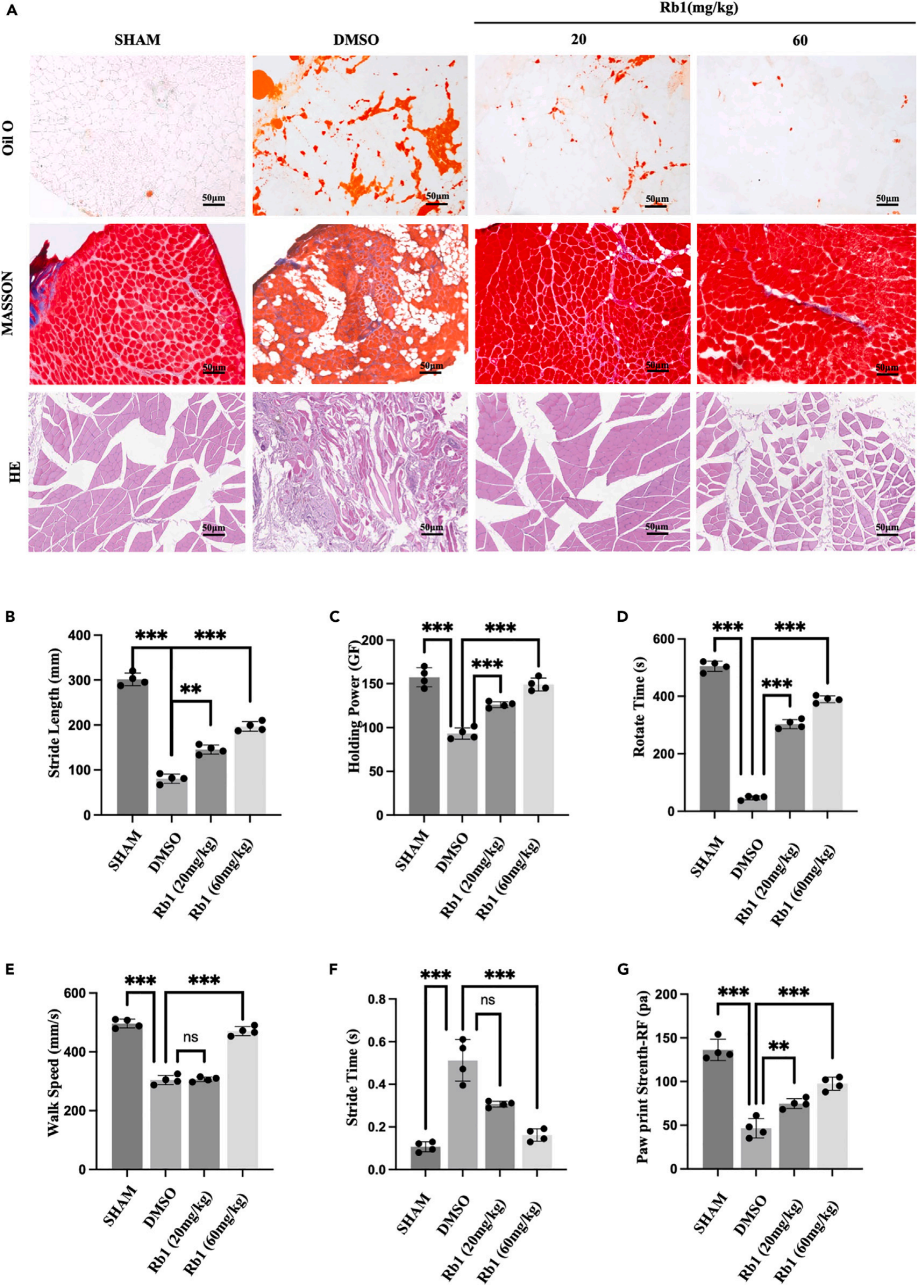
Rb1 inhibits the fat infiltration of SS muscle while improving shoulder joint function in TT modeling mice Histological and gait analysis of the SS muscle in TT modeling mice treated with Rb1 in different concentrations. (A) Oil Red O, Masson Trichrome, and H&E staining of SS muscle in TT mouse treated with different Rb1 concentrations (20 mg/kg as low concentration group and 60 mg/kg as high concentration group). (B) Measurement of stride length in walking, showing the right foot stride length. (C) Measurement of holding power, showing the holding ability of the right foot. (D) Measurement of time in the rotary test, showing the balance ability of the mouse. (E) Measurement of walk speed. (F) Measurement of stride time. (G) Measurement of paw print strength of the right foot. Scale bars: 50μm. ∗*p* < 0.05, ∗∗*p* < 0.01, ∗∗∗*p* < 0.001.

To investigate the effects of different concentrations of ginsenoside Rb1 on the supraspinatus muscle FI, we administered varying concentrations (10, 20, 40, 60, 100 mg/kg) via intraperitoneal injection in C57 mice and measured the blood drug concentrations (Figure S4C). We found that the blood drug concentration of ginsenoside Rb1 in the 10 mg/kg group was too low, while there was minimal difference between the 40 mg/kg and 100 mg/kg groups compared to the experimental group. Therefore, we ultimately decided on 20 mg/kg and 60 mg/kg as the therapeutic concentrations for the mouse experiments in this study. FI and fibrosis in the SS muscle of mice were significantly decreased in the 60 mg/kg Rb1 group compared to those in the 20 mg/kg group (Figures 2A, S3A, and S3B). Simultaneously, gait analysis showed obvious improvements in the upper limb function of the mice in the high-concentration group (Figures 2B–2G). These results indicated that ginsenoside Rb1 decreased FI in the SS muscle and improved shoulder joint function, and high concentrations had better treatment effects than low concentrations.

### Rb1 inhibits supraspinatus muscle fat infiltration by promoting lipolysis

**Figure 3:**
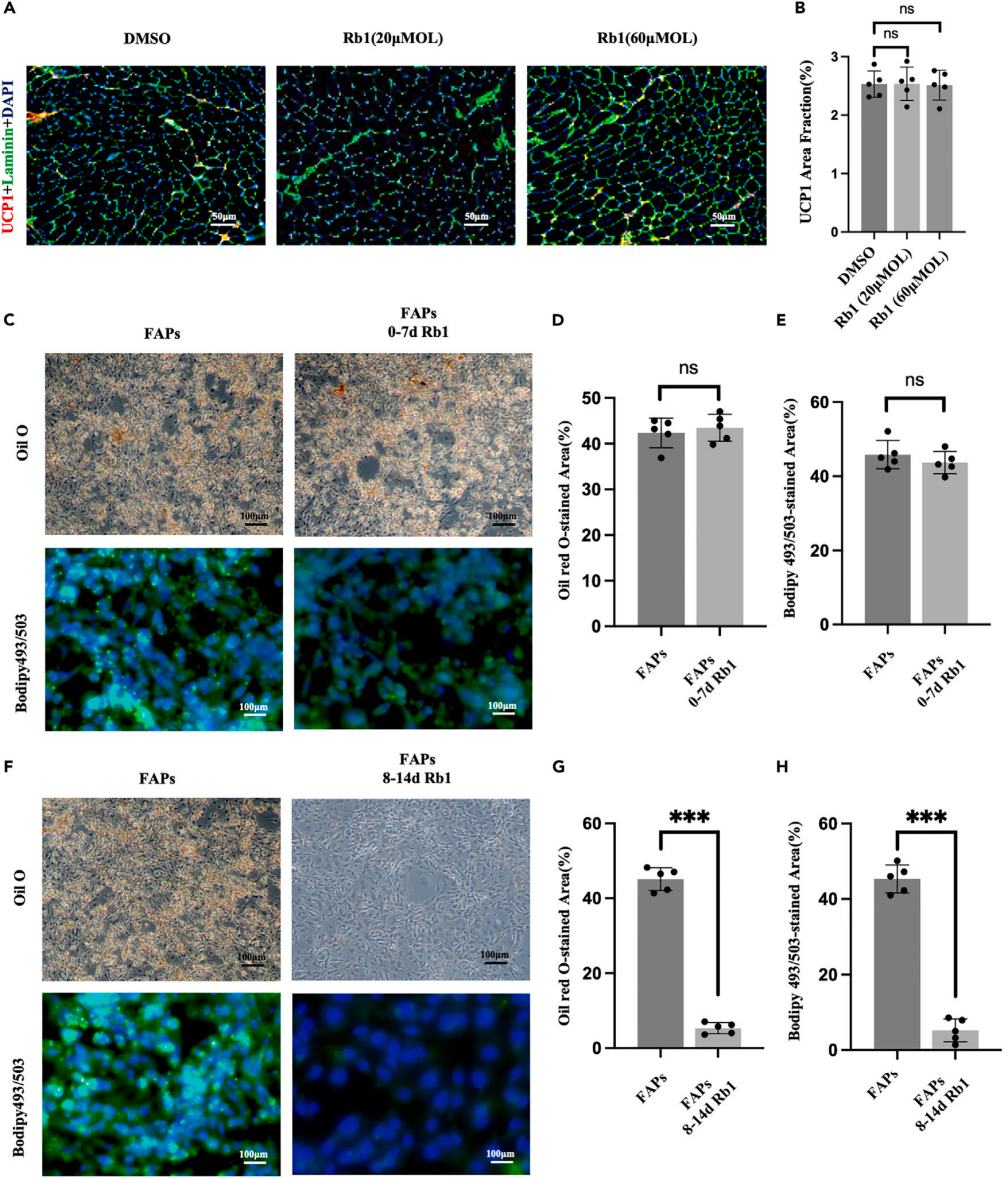
Rb1 reduces cellular fat accumulation by intervening in lipolysis (A) Expression of UCP1(red) in SS muscle of TT mouse treated with Rb1 examined by Immunofluorescence. Scale bars: 50μm. (B) UCP1 area fraction (%) = area of UCP1(red)/entire sample area. (C–E) Oil O and Bodipy 493/503 staining of fibro-adipogenic progenitors (FAPs) treated with Rb1 at the early stage (0-7days) of adipogenic differentiation. (F–H) Oil O and Bodipy 493/503 staining of FAPs treated with Rb1 at the late stage (8-14days) of adipogenic differentiation. ∗∗∗*p* < 0.001.

Next, we determined the molecular mechanisms underlying the effects of Rb1 on intramuscular FI after injury. Pathological FI is always caused by abnormal lipid metabolism such as lipolysis, adipogenesis, and adipose differentiation.^18^^,^^19^ UCP1 is a key molecule in brown adipose tissue and is a marker of adipose differentiation.^20^ The results of immunofluorescence staining have shown that there was no statistical difference in the expression of UCP1 between mice undergoing Rb1 intervention and the control group (Figures 3A and 3B). Next, to study the effect of Rb1 on lipolysis and lipogenesis, mouse fibro/adipogenic progenitors (FAPs) were obtained from the SS muscles of mice by flow sorting (Figure S4A). FAPs are PDGFRα^+^ cell populations in the rotator cuff muscle group which have the potential to differentiate into adipose tissue and fibrous tissue.^21^ Pathological adipogenesis of FAPs has been reported to be the main source of FI in skeletal muscle.^22^^,^^23^^,^^24^ When FAPs were cultured in an adipogenic differentiation medium, the cells turned into white adipose cells after 7 days (Figures S4B and S4C). Rb1 was added on the first day or 7 days after FAPs were cultured in adipogenic differentiation medium for one week to determine whether Rb1 intervened in adipogenesis or lipolysis. Oil Red O and BODIPY 493/503 staining were used to detect intracellular lipid changes. The results showed that, compared to the control group, there was no statistical difference of FAPs cells in the size and number of intracellular lipid droplets in the 0–7 days Rb1 group (Figures 3C–3E). Conversely, we found that the size and number of intracellular lipid droplets in the 7–14 days Rb1 group were significantly reduced when compared to the control group (Figures 3F–3H). In conclusion, Rb1 promoted the fatty acid decomposition of adipocytes in the rotator cuff muscle to decrease intramuscular FI after fracture.

### Rb1 regulates CPT1A deacetylation to promote fatty acid β-oxidation

**Figure 4:**
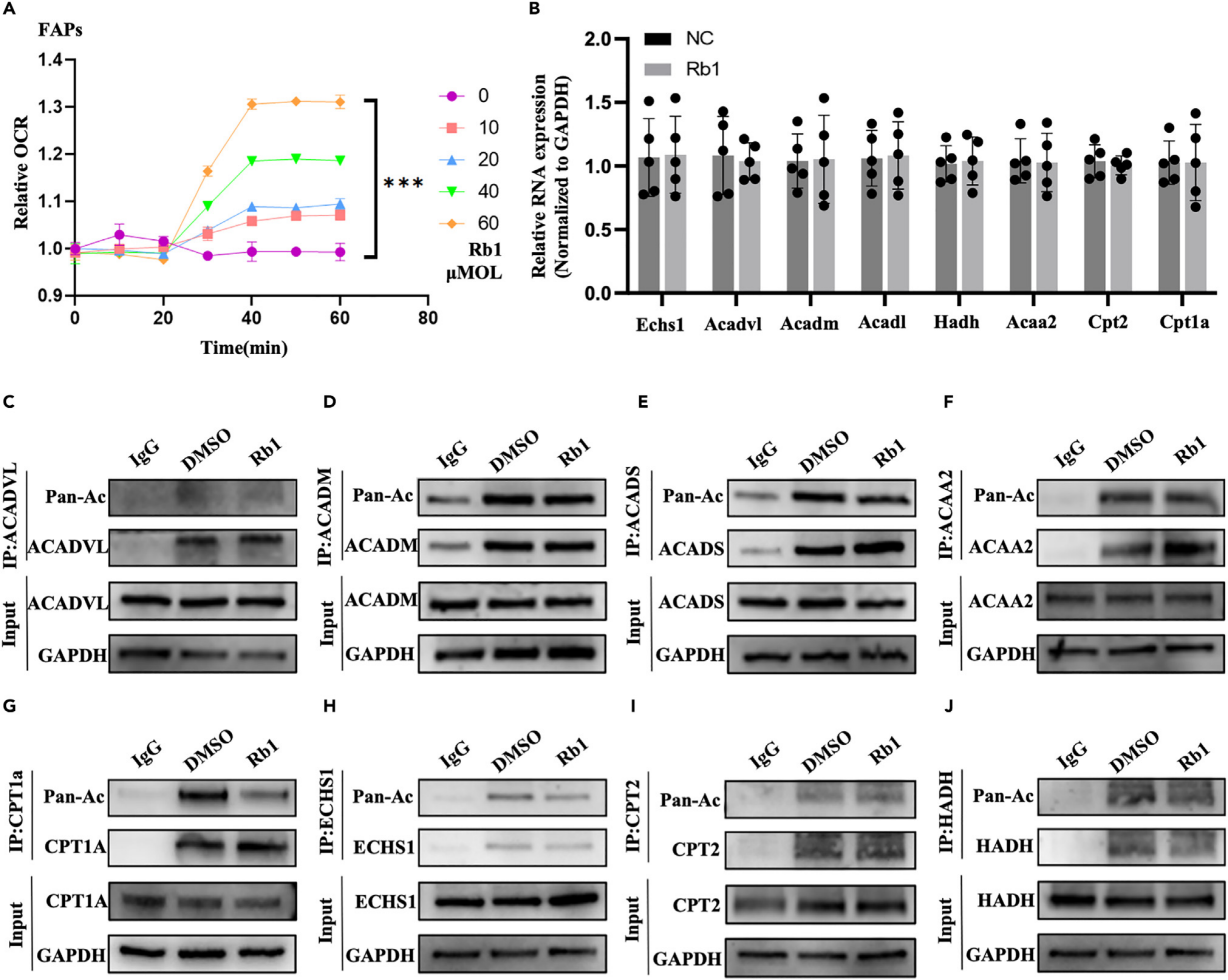
Rb1 promotes lipolysis by regulating CPT1A acetylation levels (A) Effects of Rb1 of different concentrations (10,20,40,60μM) on real-time mitochondrial oxygen consumption rate of FAPs cells. (B) Expression of β-oxidation genes (Echs1, Acadvl, Acadm, Acadl, Hadh, Acaa2, Cpt2, Cpt1a) were detected by RT-qPCR in 3T3-L1 cells treated with Rb1 or DMSO. (C–J) The protein expression and acetylation level of the above fatty acids β-oxidation genes were detected by immunoprecipitation and Western blot in 3T3-L1 cells treated with Rb1 or DMSO.

Lipolysis is a process in which triglycerides are decomposed into fatty acids and glycerol and can either be released by cells into the extracellular matrix or enter the tricarboxylic acid cycle for heat production.^25^ We measured oxygen consumption by FAPs after adipogenic differentiation induction with different concentrations of Rb1. We found that the oxygen consumption of the cells increased with an increase in Rb1 concentration (Figure 4A). To further study the molecular mechanism of the effect of Rb1 on lipolysis, we detected the mRNA and protein expression levels of key fatty acid β-oxidative enzymes (Echs1, Acadvl, Acadm, Acadl, Hadh, Acaa2, Cpt2, Cpt1a) using quantitative PCR (qPCR) and Western blot (WB) analysis in 3T3-L1 cells. The results have shown that their mRNA and protein expression levels did not change significantly (Figure 4B). Some studies have reported that acetylation is involved in various stages of fatty acid β-oxidation.^26^^,^^27^^,^^28^ Therefore, we further explored whether Rb1 changes the acetylation levels of the key enzyme of β-oxidation to affect lipolysis. Interestingly, we found that CPT1A was significantly deacetylated after Rb1 treatment, accompanied by no changes in other enzymes (Figures 4C–4J). CPT1A is a transmembrane transporter located in the mitochondrial membrane Its main role is to convert long-chain fatty acids into fatty acylcarnitine and transport them to mitochondria to participate in subsequent fatty acid β-oxidation reaction.^29^ We supposed Rb1 may modify CPT1A deacetylation, resulting in the activation of fatty acid β-oxidation.

### Rb1 regulates CPT1A deacetylation via SIRT1

**Figure 5:**
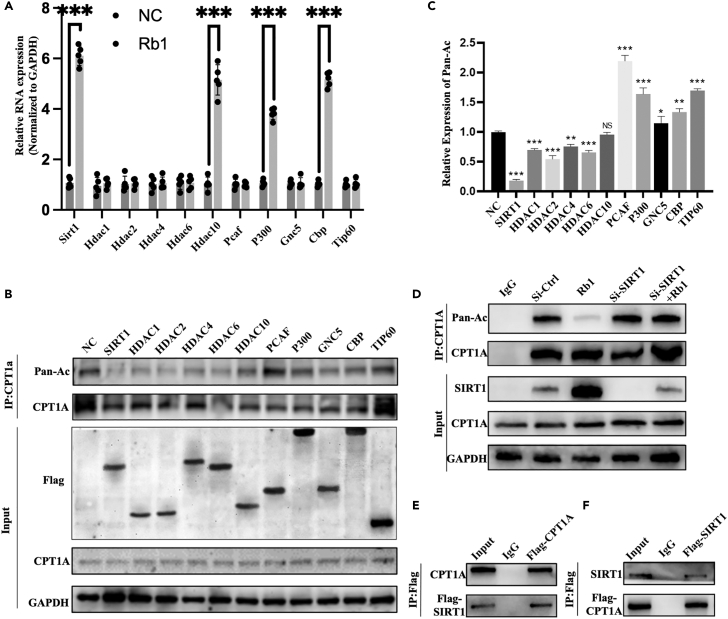
SIRT1 is the key enzyme regulating the acetylation level of CPT1A (A) After treating 3T3-L1 cells with 60μM Rb1 for 1 week, expression of KAT (Pcaf, p300, Gnc5, Cbp, Tip60) and KDAC (Sirt1, Hdac1, Hdac2, Hdac4, Hdac6, Hdac10) genes were detected by RT-qPCR. (B and C) The expression and acetylation level of CPT1A was detected in 3T3-L1 cells after overexpressed with the above KAT and KDAC genes. (D) The expression and acetylation level of CPT1A was detected in 3T3-L1 treated with Rb1 or Rb1 combined with Si-SIRT1. (E and F) Co-immunoprecipitation assay detected interaction between SIRT1 and CPT1A. ∗*p* < 0.05, ∗∗*p* < 0.01, ∗∗∗*p* < 0.001.

**Figure 6:**
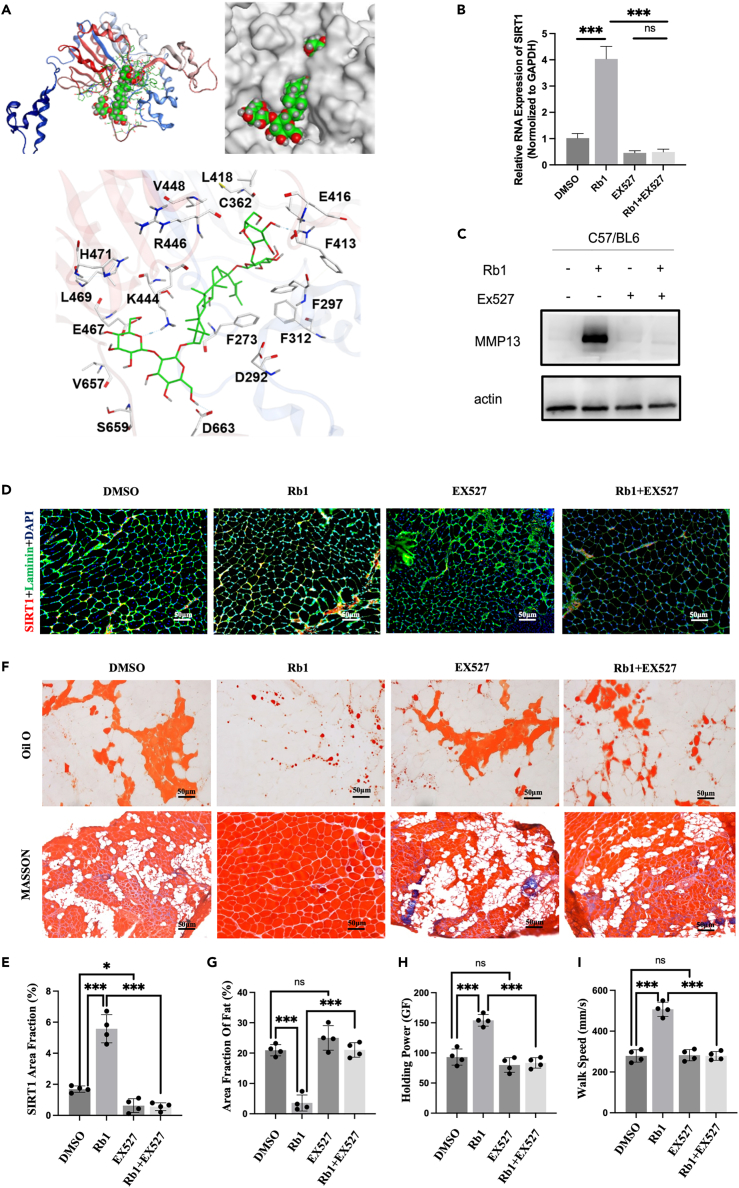
Rb1 ameliorates SS muscle fat infiltration by binding to SIRT1 (A) Molecular docking assay of Rb1 with the NAD^+^ binding site of SIRT1(PDB ID 4ZZJ), the affinity between ginsenoside Rb1 and Sirt1 is −12.27 kcal/mol, and the pentose sugar moiety of the compound forms hydrogen bond interactions with the amino acids F413, K444, E467, and D663 of Sirt1. (B) SIRT1 RNA Expression of rotator cuff in TT mouse treated with Rb1 and EX527 separately and together; (C)SIRT1 protein expression of rotator cuff in TT mouse treated with Rb1 and EX527 separately and together; (D) Expression of SIRT1 (red) examined by Immunofluorescence in SS muscle of TT mouse treated with Rb1 and EX527 separately and together. Scale bars: 50μm. (E) Oil Red O and Masson Trichrome staining of SS muscle in TT mouse treated with Rb1 and EX527 separately and together. Scale bars: 50μm. (F) SIRT1 area fraction = area of SIRT1(red)/entire sample area. (G) Fat area fraction analysis of Oil Red O staining. (H and I) Measurement of holding power and walk speed of TT mouse treated with Rb1 and EX527 separately and together. ∗∗∗*p* < 0.001.

To further explore the specific mechanism by which Rb1 regulates the acetylation levels of CPT1a, we examined the mRNA levels of lysine acetyltransferases (KATs) (*Pcaf*, *P300*, *gnc5*, *Cbp*, and *Tip60*) and lysine deacetylases (KDACs) (*Sirt1*, *Hdac1*, *Hdac2*, *Hdac4*, *Hdac6*, and *Hdac10*) after 3T3-L1 cells were treated with Rb1. *Sirt1*, *Hdac10*, *P300*, and *Cbp* were upregulated after Rb1 treatment (Figure 5A). Next, we overexpressed these enzymes in 3T3-L1 cells and detected changes in the acetylation levels of CPT1A. We found that SIRT1, HDAC2, HDAC4, and HDAC6 could downregulate the acetylation levels of CPT1A, whereas PCAF and Tip60 could upregulate these levels (Figures 5B and 5C). Only SIRT1 was regulated by Rb1, which in turn regulated the acetylation levels of CPT1A. The regulation of CPT1A deacetylation by Rb1 was reversed by SIRT1 knockdown (Figures 5D and S5A). Therefore, we focused on the function of SIRT1. Co-immunoprecipitation (Co-IP) showed that SIRT1 could bind to CPT1A (Figures 5E and 5F). Additionally, SIRT1 expression levels increased in 3T3-L1 cells following treatment with increasing concentrations of Rb1 (Figure S5B). Using the Molecular Operating Environment software, we first predicted the affinity of Rb1 binding to the three binding domains of SIRT1 and found that the NAD^+^ binding site had the strongest affinity for Rb1 (Figure S6). Using a flexible docking method, we constructed a binding pattern map of Rb1 and the NAD^+^ binding site of SIRT1. We found that Rb1 was embedded in the interior of SIRT1, and multiple amino acids of SIR1 may be involved in its interaction with Rb1 (Figures 6A and S7). Thus, we inferred that Rb1 may promote SIRT1 activation by binding to its NAD^+^-binding domain and deacetylating CPT1A.

After the transfection of si-SIRT1 into 3T3-L1 cells treated with Rb1, the size and number of lipid droplets inhibited by Rb1 increased (Figure S9B). Furthermore, the TT mice were treated with Rb1 and EX527 (a SIRT1 specific inhibitor). We found that Rb1 enhanced SIRT1 expression in the SS muscle, and EX527 reversed this change (Figures 6B–6E). FI and fibrosis in the SS muscle of mice were significantly increased in the Rb1 + EX527 group compared to those in the Rb1-only treatment group (Figures 6F, 6G, and S9A–S9D). Moreover, the improvement in shoulder joint function induced by Rb1 was reversed by EX527 treatment (Figures 6H, 6I, and S9G–S9L). These results suggested that Rb1 deacetylates CPT1A by binding to and activating SIRT1 to decrease intramuscular FI and improve joint function after injury.

### Rb1 regulates lipolysis by inducing CPT1A deacetylation at the K195 site

**Figure 7:**
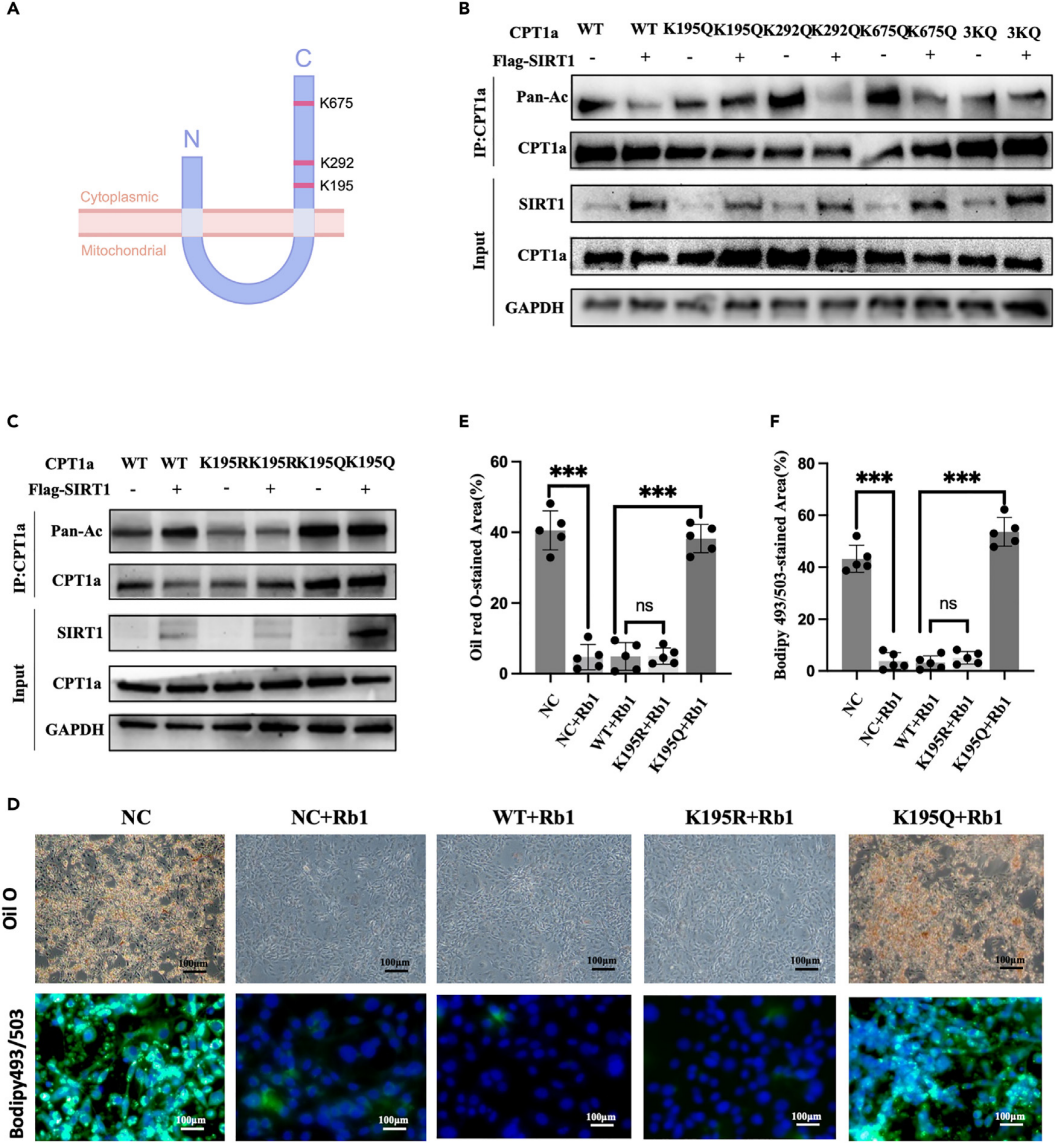
Rb1 regulates lipolysis via affecting the CPT1A deacetylation at K195 site (A) Schematic representation of the SIRT1 in the mitochondrial outer membrane with the 195,292,675 lysine residue. (B) 3T3-L1 cells were transfected with Flag-SIRT1 and wild type CPT1A (WT) or the different lysine-to-arginine mutants (K195Q, K292Q, K675Q, 3KQ) together. Acetylation level of CPT1A was detected by immunoprecipitation and Western blot using a pan-acetylation antibody. (C) 3T3-L1 was transfected with Flag-SIRT1 and CPT1A WT or the k195Q or 195 lysine-to-glutamine (k195R) mutants together. Acetylation level of CPT1A was detected by immunoprecipitation and Western blot using the pan-acetylation antibody. (D–F) Oil O and Bodipy 493/503 staining of 3T3-L1 cells transfected with CPT1A WT or the K195R or K195Q mutants and treated with Rb1. ∗∗∗*p* < 0.001.

There were three potential sites for the deacetylation of CPT1A: K195, K292, and K675 (Figure 7A). We constructed mutant plasmids in which each of the three lysine residues was changed to arginine (Q) to simulate the acetylated state of CPT1A. We found that only in CPT1A^K195Q^ or CPT1A^3KQ^ did the acetylation level of CPT1A in 3T3-L1 cells not change upon SIRT1 overexpression (Figures 7B and S10A). We constructed the mutant plasmid CPT1A^K195R^ to simulate the deacetylated state of CPT1A, in which the lysine residue was converted to arginine (R). Similarly, with CPT1A^K195Q^, deacetylation of the CPT1A^K195R^ plasmid was not affected by SIRT1 (Figures 7C and S10B). Finally, we found that the decrease in intracellular lipid levels induced by Rb1 in CPT1A^wt^ 3T3-L1 cells was maintained in CPT1A^K195R^ cells and abolished in CPT1A^K195Q^ cells (Figures 7D–7F). Therefore, we speculated that the deacetylation of K195 in CPT1A, influenced by Rb1 through SIRT1, was the main reason for the change in its enzymatic activity to promote lipolysis.

## Discussion

RCTs are a common shoulder joint injury. If left untreated in a timely manner, they can lead to muscle degeneration including FI. Previous clinical studies have found that muscle degeneration, such as FI of the rotator cuff muscles, is closely associated with the prognosis of shoulder joint function and pain. Therefore, treating FI of the rotator cuff muscles is an important clinical issue in the management of RCTs and their complications.^30^^,^^31^^,^^32^ However, there is no effective way to inhibit FI in the rotator cuff muscles. The present study found that treatment with ginsenoside Rb1 decreased FI in the SS muscle by enhancing lipolysis. Furthermore, we clarified that Rb1 could bind to the NAD^+^ binding domain of SIRT1 with high affinity which increased the deacetylation of CPT1A in the lysine 195 site to promote fatty acid β-oxidation.

Increasing attention has been directed toward the impact of Ginsenoside Rb1 on lipid metabolism. Ginsenoside Rb1 has been shown to promote lipolysis and facilitating the browning of adipose tissue in db/db mice.^33^ Furthermore, studies have indicated that in hepatic steatosis, Ginsenoside Rb1 could activate mitochondrial β-oxidation functions and ameliorate fatty liver by regulating some key enzyme expression and CPT1A active.^34^ Our study found Rb1 increased the oxygen consumption of FAPs and Rb1 decreased FI in muscle after injury by promoting lipolysis in FAPs. We also detected key fatty acid β-oxidative enzymes in our model. However, the mRNA and protein expression levels of these genes were almost unchanged during this process. Through further exploring, we found only the CPT1A protein deacetylated modification was significantly increased after Rb1 treatment.

Protein acetylation in the human body is regulated by KAT and KDAC. Therefore, we examined changes in their expression after Rb1 treatment and found that only SIRT1 expression was influenced by Rb1 treatment. Ginsenoside Rc has been reported to act as an agonist of SIRT1 in a previous study,^35^ however it remains unknown how Rb1 activates SIRT1. We used software to predict whether Rb1 could bind to the NAD^+^ binding domain of SIRT1 by embedding and forming hydrogen bonds. Furthermore, both *in vitro* and *in vivo* rescue examinations showed that SIRT1 is essential for the therapeutic effect of Rb1 on FI. Therefore, we hypothesized that Rb1 promotes CPT1A deacetylation via SIRT1 to enhance the fatty acid β-oxidation of adipose cells and decrease intramuscular FI after injury. Unfortunately, we did not explore the detailed mechanism of Rb1 activation by SIRT1 in this study. This will investigated using surface plasmon resonance, isothermal calorimetric titration, and microthermal surge experiments in the future.

CPT1A is a monomeric channel protein that spans the mitochondrial membrane twice. Its N48-M122 sites are the transmembrane and inner mitochondrial membrane regions, and the other domains are located in the cytoplasm, forming a catalytic domain (residues 123–773).^36^ In channel proteins, post-translational modifications may lead to changes in their conformation or degree of multimer binding, thereby causing changes in their function.^37^ CPT1A can homo-oligomerize to form trimers, which are essential for channel function.^38^ We found that the K195 deacetylation site of CPT1A, which is regulated by SIRT1, is close to the mitochondrial membrane. We speculated that the deacetylation of K195 may lead to changes in the catalytic domain or homo-oligomerization state of CPT1A, which increases the conversion of fatty acids to lipoyl coenzyme A promoting fatty acid transport into the mitochondria. However, this hypothesis warrants further investigation.

Additionally, our study has several limitations: we did not investigate the specific mechanisms by which acetylation changes at specific sites affect the function of the CPT1a molecule. Moreover, our molecular investigations were confined to *in vitro* studies, without extending to *in vivo* animal experiments. Furthermore, we did not conduct gait trajectory analysis or other assessments to reflect changes in shoulder joint function in mice. We plan to address these limitations with further experimental validation in future research.

In conclusion, we verified that ginsenoside Rb1 can deacetylate and modify the lysine 195 site of CPT1A by binding to and activating SIRT1, thereby activating fatty acid β-oxidation and lipolysis to inhibit FI in the rotator cuff muscles and improve shoulder joint function after injury. In the future, we will explore the potential of ginsenoside Rb1 for clinical use and its therapeutic effects.

## STAR★Methods

### Key resources table

**Table:** 

REAGENT or RESOURCE	SOURCE	IDENTIFIER
**Antibodies**		

Rabbit polyclonal anti-SIRT1	Proteintech	Cat# 13161-1-AP; RRID: AB_10646436
Rabbit polyclonal anti-ACADVL	Proteintech	Cat# 14527-1-AP; RRID: AB_2288885
Rabbit polyclonal anti-ACADM	Proteintech	Cat# 55210-1-AP; RRID: AB_10837361
Rabbit polyclonal anti-ACADS	Proteintech	Cat# 16623-1-AP; RRID: AB_10666165
Rabbit polyclonal anti-CPT1A	Proteintech	Cat# 15184-1-AP; RRID: AB_2084676
Rabbit polyclonal anti-ACAA2	Proteintech	Cat# 11111-1-AP; RRID: AB_2219389
Rabbit polyclonal anti-vECHS1	Proteintech	Cat# 11305-1-AP: RRID: AB_2262166
Rabbit polyclonal anti-CPT2	Proteintech	Cat# 26555-1-AP; RRID: AB_2880551
Rabbit polyclonal anti-HADH	Proteintech	Cat# 19828-1-AP; RRID: AB_10667408
Rabbit polyclonal anti-GAPDH	Proteintech	Cat# 60004-1-Ig; RRID: AB_2107436
Rabbit polyclonal anti-pan acetylation	Proteintech	Cat# 66289-1-Ig; RRID: AB_2881672
Rabbit polyclonal anti-FLAG	Abcam	Cat# ab205606; RRID: AB_2916341
Rabbit polyclonal anti-UCP1	Proteintech	Cat# 23673-1-AP; RRID: AB_2828003
Rabbit polyclonal anti-laminin	Proteintech	Cat# 23498-1-AP; RRID: AB_2879288
anti-CD31-fluorescein isothiocyanate	BD Biosciences	Clone 390
anti-CD45-FITC	BD Biosciences	Clone WM59
anti-integrin α7-APC	R&D Systems	Clone #334908l
PE-Cy7-Sca1	BD Biosciences	Clone E13–161.7

**Chemicals, peptides, and recombinant proteins**		

Ginsenoside Rb1	MCE	Cat# 41753-43-9
Ginsenoside Rc	G-clone	Cat# 11021-14-0
Ginsenoside Rd	G-clone	Cat# 52705-93-8
Ginsenoside Rf	G-clone	Cat# 52286-58-5
Ginsenoside Rg2	G-clone	Cat# 52286-74-5
EX527	MCE	Cat# HY-15452
Oil Red O Saturated solution	Solarbio Science & Technology Co	G1260
Modified Masson's trichrome staining kit	Solarbio Science & Technology Co	G1346
Hematoxylin-Eosin (HE) Stain Kit	Solarbio Science & Technology Co	G1120
mounting medium containing DAPI	Biosharp	BL739A
BODIPY staining solution	GlpBio	GC42959

**Experimental models: Cell lines**		

Mouse: 3T3-L1 cell line	Procell	CL-0006

**Experimental models: Organisms/strains**		

Mouse: 110 female C57/BL6	Hunan SJA Company, China	2019sydw0198

**Oligonucleotides**		

Primers for Echs1, See Table S1	This Paper	N/A
Primers for Acadvl, See Table S1	This Paper	N/A
Primers for Acadm, See Table S1	This Paper	N/A
Primers for Acadl, See Table S1	This Paper	N/A
Primers for Hadh, See Table S1	This Paper	N/A
Primers for Acaa2, See Table S1	This Paper	N/A
Primers for Cpt2, See Table S1	This Paper	N/A
Primers for Cpt1a, See Table S1	This Paper	N/A
Primers for Sirt1, See Table S1	This Paper	N/A
Primers for Hdac1, See Table S1	This Paper	N/A
Primers for Hdac2, See Table S1	This Paper	N/A
Primers for Hdac4, See Table S1	This Paper	N/A
Primers for Hdac6, See Table S1	This Paper	N/A
Primers for Hdac10, See Table S1	This Paper	N/A
Primers for PCAF, See Table S1	This Paper	N/A
Primers for P300, See Table S1	This Paper	N/A
Primers for Gnc5, See Table S1	This Paper	N/A
Primers for Cbp, See Table S1	This Paper	N/A
Primers for Tip60, See Table S1	This Paper	N/A

**Recombinant DNA**		

CPT1AK195Q, CPT1AK195R, CPT1AK262Q, CPT1AK675Q, CPT1A3KQ mutant, Flag-CPT1A, and Flag-SIRT1 plasmids	Hunan Fenghui Biotechnology Co., Ltd.	N/A
Plasmids of KAT (Pcaf, P300, gnc5, Cbp, and Tip60) and KDAC (Sirt1, Hdac1, Hdac2, Hdac4, Hdac6, and Hdac10)	Dr. Kang Tiebang of SYSUCC	

**Software and algorithms**		

ImageJ	National Institute of Health, Bethesda	1.48V
Gait analysis software	(Beijing Kangsen Yi You Bio-Tech Co	N/A
Molecular Operating Environment software	Chemical Computing Group ULC	ver. 2022.02
GraphPad Prism 9	GraphPad	N/A
SPSS ver. 25	IBM	N/A

**Other**		

40-μm cell strainer	corning	431750
70-μm cell strainer	corning	431751
Oxygen Consumption Rate Assay Kit	Cayman Chemical	600800
HiScript II Q RT SuperMix for qPCR (+gDNA wiper)	Vazyme	R223-01
jetPRIME	PolyPlus	101000046

### Resource availability

#### Lead contact

Further information and requests for resources and reagents should be directed to and will be fulfilled by the lead contact, Yuesong Yin (2204160408@csu.edu.cn).

#### Materials availability

This study did not generate new unique reagents, plasmids and animal lines.

#### Data and code availability

• Data reported in this paper will be shared by the lead contact upon request.
• This paper does not report original code.
• Any additional information required to reanalyze the data reported in this work paper is available from the lead contact upon request.

### Experimental model and study participant details

#### Ethics

Animal experiments were conducted in accordance with the Guide for the Care and Use of Laboratory Animals published by the National Institutes of Health and were approved by the Institutional Animal Care and Use Committee of Central South University (No.2019sydw0198). Extensive efforts have been made to minimize animal suffering.

#### Animals

Six-week-old female C57/BL6 mice (n=110) were housed in cages with a 12-h dark-light cycle, free access to water, and a regular chow diet for one week to allow acclimatization to animal care facilities. To construct mouse model of TT, the right-side SS muscle tendon in the mice was transected completely after general anesthesia was achieved via 1–5% isoflurane inhalation. In the sham group, skin and muscle incisions were made, the SS tendon was exposed, and the muscles and skin were closed. For the ginsenoside treatment groups (n=5 per group), ginsenosides Rb1, Rc, Rd, Rf, and Rg2 (G-clone, China) were dissolved in 0.1 mL of 1% DMSO and administered to mice via intraperitoneal injection, starting on the same day of surgery and repeated five days per week. The same volume of 1% DMSO was administered to mice in the vehicle control group (n=5). Mice were euthanized by carbon dioxide inhalation 6 weeks after surgery. For the different concentration groups (n=10 per group), we treated TT model mice with Rb1 (purity = 98.75%; MCE, China) at high (60 mg/kg) and low (20 mg/kg) concentrations, a sham surgery group as the negative control (n=10), and a group of mice treated with 0.1 mL 1% DMSO as the positive control group (n=10). For rescue examination (n=10 per group for four groups), 30 mg/kg EX527 (HY-15452; MCE) was introduced to inhibit the expression of SIRT1. To further explore the mechanism of SIRT1 and Rb1, we divided the mice into four groups after TT modeling: DMSO treatment, Rb1 treatment, EX527 treatment, and Rb1 + EX527 treatment. In each group, five C57B/L6 mice were used for histological analysis and the other five mice were used for gait analysis.

#### Muscle digestion and FAP isolation

Two weeks after TT, FAPs were extracted from the SS muscles of C57/BL6 wild-type mice (n=4). SS muscles from the surgical and sham sides were minced into 1-mm tiny pieces in a cell culture hood. In a sterile water bath heated to 37°C for 90 min, the mixture was incubated with 0.2% collagenase II. The mixture was mixed with 40 mL of washing buffer (F/10, 10% fetal bovine serum [FBS], and 1 mL HEPES), and then centrifuged at 1500 rpm for 5 min at 25°C. The supernatant was then poured into a new 50 mL centrifuge tube. The residue was centrifuged at 1500 rpm for 5 min after washing with wash buffer. The supernatant was collected and combined with that obtained in the previous round. The supernatant was mixed with the supernatant obtained in the previous round. The D2 solution (0.06% collagenase II and 0.15% dispase D with wash buffer) was incubated for 30 min at 37°C. Next, the solutions were run through a 40-μm cell strainer (VWR International, Radnor, PA, USA) and a 70-μm cell strainer (VWR International). The filtered cells were then centrifuged at 1500 rpm for 5 min after washing with 40 mL fluorescence-activated cell sorting (FACS) buffer (2.5% FBS, 20 mM EDTA, and PBS). The cell pellets were resuspended in 500 mL FACS buffer after the supernatant was removed and discarded. Cells were incubated with anti-CD31-fluorescein isothiocyanate (FITC; Clone 390; BD Biosciences, Franklin Lakes, NJ, USA), anti-CD45-FITC (Clone WM59; BD Biosciences), anti-integrin α7-APC (Clone #334908l; R&D Systems, Minneapolis, MN, USA), and PE-Cy7-Sca1 (Clone E13–161.7; BD Biosciences) for 30 min before sorting with FACS Aria™ II (BD Biosciences). FAPs were collected as CD31^−^/CD45^−^/ITGA7^−^/Sca1^+^ cell populations.

#### Cell culture

The FAPs were grown in adipogenic differentiation medium (100 μM IBMX, 2.5 μM DEX, 100 mM indomethacin, and 10 μg/mL insulin). When FAPs were cultured in adipogenic differentiation medium, they turned into white adipose cells within 7 days.^39^^,^^40^ Rb1 was added on the first day or 7 days after the FAPs were cultured in adipogenic differentiation medium lasting for one week. 3T3-L1 cells were purchased from Procell (Procell Life Science & Technology Co., Ltd., China). Standard cell culture medium (SM; Ham's F-10, 10% FBS, 10 ng/mL basic fibroblast growth factor , and 1% antibiotic-antimycotic solution; Thermo Fisher Scientific, Waltham, MA, USA) was used to cultivate 3T3-L1 cells. Rb1 was added 7 days after 3T3-L1 cells were cultured in adipogenic differentiation medium (100 μM IBMX, 2.5 μM DEX, 100 mM indomethacin, and 10 μg/mL insulin) lasting one week to investigate the role of SIRT1 in adipose metabolism.

### Method details

#### Histological analysis

SS muscles of C57B/L6 mice in different groups (n=5 per group) were flash-frozen and cryosectioned. An Oil Red O Saturated solution (G1260; Beijing Solarbio Science & Technology Co., Ltd., China) and double-distilled water were mixed at a ratio of 3:2 to prepare the working solution. The slides were stained with Oil Red O working solution for 20 min and covered with 10% glycerol in phosphate-buffered saline (PBS) before observation. Masson’s trichrome staining was performed using a Modified Masson's trichrome staining kit (G1346; Beijing Solarbio Science & Technology Co., Ltd., China) to measure fibrosis in the SS muscle. The slides were stained with Ponceau acid fuchsin staining buffer for 3 h, aniline blue staining buffer for 2 min, and covered with 50% xylene resin. The slides were observed under a light microscope. Images were examined using ImageJ (1.48V) software (National Institute of Health, Bethesda, MD, USA).

#### Grip strength tests and gait analysis

Briefly, mice (n=5 per group) were placed on a long glass track after training, and a camera was used to record the deformation of the track when the mice stepped on the glass track. An automated computer-assisted method (Beijing Kangsen Yi You Bio-Tech Co. Ltd., China) was used for the analysis. This study analyzed the walking speed, paw print strength, stride time, stride length, holding power, and rotation time of the mice.

#### Immunofluorescence staining

The sections were washed with water after fixation with a 4% PFA Fix Solution. Sections were blocked with BSA solution for 1 h, incubated with SIRT1 (1:3000; Proteintech, Rosemont, IL, USA), UCP1 (1:1000; Proteintech), and laminin antibodies (1:2000; Proteintech) for 16–20 h. The sections were then incubated with a fluorescent secondary antibody for 1 h in the dark. After washing, the slices were sealed with antifade mounting medium containing DAPI (Bioshark, China) and observed under a fluorescence microscope. The area fraction of immunofluorescence was measured using the ImageJ software.

#### Oil Red O and BODIPY 493/503 staining of cells

Prior to cell seeding, 24-well cell plates were coated for 1 h at 37°C with 1% Matrigel in Dulbecco's modified Eagle's medium. After sorting, 5000 cells/well of 24-well plates were seeded with FAPs or 3T3-L1 cells. After fixation, the cells were submerged in BODIPY staining solution (2 μM, GC42959; GlpBio, Montclair, CA, USA) or filtered Oil Red O stain (ORS; Solarbio, China) working solution for 15 min at 37°C in the dark. After discarding the dye solution, the adipocytes were washed twice with double-distilled water. Cell images were captured and examined using an inverted microscope. Lipid droplet sizes were measured using ImageJ software.

#### Oxygen consumption rate

An Oxygen Consumption Rate Assay Kit (600800; Cayman Chemical, Ann Arbor, MI, USA) was used to examine the oxygen consumption rate of the FAPs. Briefly, after FAPs were treated with different concentrations of Rb1, they were incubated with Phosphorescent Oxygen Probe Solution, and then measured with SpectraMAX at 37°C. Plates were read every 10 min for 60 min.

#### Real-time qPCR

TRIzol reagent was used to extract total RNA from the muscles and cells in accordance with the manufacturer's instructions. cDNA was produced using HiScript II Q RT SuperMix for qPCR (+gDNA wiper) (R223-01; Vazyme, China). Following the manufacturer's instructions, ChamQ Universal SYBR qPCR Master Mix (Vazyme) was used for real-time qPCR under the following parameters; 40 cycles of denaturation at 95°C for 10 s, annealing at 56°C for 30 s, and extension at 72°C for 30 s after 15 min of initial denaturation at 95°C. Table S1 lists the primer sequences for the target genes. For muscle, the expression levels were normalized to those of S26, a popular housekeeping gene that is constitutively expressed in mouse muscle. And for adipose tissue, the expression levels were normalized to those of GAPDH. Fold changes relative to the sham group were calculated using ΔΔCT.

#### WB analysis and IP

RIPA buffer was used for whole-cell extracts from 3T3-L1 cells before WB analysis. The following antibodies were used: SIRT1 (1:3000; Proteintech), ACADVL (1:2000; Proteintech), ACADM (1:3000; Proteintech), ACADS (1:2000; Proteintech), CPT1A (1:5000; Proteintech), ACAA2 (1:2000; Proteintech), ECHS1 (1:2000; Proteintech), CPT2 (1:2000; Proteintech), HADH (1:3000; Proteintech), GAPDH (1:5000; Proteintech), pan acetylation (1:1000; Proteintech), and FLAG (1:3000; Abcam). For WB analysis, 5× SDS-PAGE loading buffer (Merck, Germany) was added to the boiled proteins, which were then immunoblotted with appropriate primary and secondary antibodies and detected by chemiluminescence. For IP analysis, cell lysates (900 μL) after pre-clearing were mixed with antibodies (2 μg) and magnate beads (Bimake, China) at 4°C overnight. The immune complexes were washed 7–10 times with Cell Lysis Buffer for WB and IP without inhibitors (NCM Biotech, Newport, RI, USA). After boiling in 5× loading buffer, the samples were subjected to SDS-PAGE and visualized using enhanced chemiluminescence.

#### Molecular docking

To investigate the interaction between Rb1 and SIRT1, molecular docking was performed using the Molecular Operating Environment software ver. 2022.02 (Chemical Computing Group ULC, Canada). Sirt1 structure (PDB ID 4ZZJ) was used as the receptor file for molecular docking with Rb1, the Triangle Match algorithm is used to generate docking poses of the complexes, while the London δG scoring function is utilized to compute the affinity of each docking pose. The top 30 docking poses with the best affinity are retained, and these poses are subsequently optimized under the Induced Fit algorithm. The GBVI/WAS δG scoring function is employed to calculate the affinity of the optimized docking poses. Based on the molecular docking interface pocket region, the five best docking poses of Sirt1 and the compound are finally retained.

#### Plasmids, siRNA, and transfection

CPT1A^K195Q^, CPT1A^K195R^, CPT1A^K262Q^, CPT1A^K675Q^, CPT1A^3KQ^ mutant, Flag-CPT1A, and Flag-SIRT1 plasmids were purchased from Hunan Fenghui Biotechnology Co., Ltd. Plasmids of KAT (*Pcaf*, *P300*, *gnc5*, *Cbp*, and *Tip60*) and KDAC (*Sirt1*, *Hdac1*, *Hdac2*, *Hdac4*, *Hdac6*, and *Hdac10*) were gifts from Dr. Kang Tiebang of SYSUCC. Transfection of plasmids and Si-SIRT1 (Ribo, China) was performed using a jetPRIME (101000046, PolyPlus, France). Cells were captured 2 days after transfection, and RNA and proteins were collected for subsequent experiments.

### Quantification and statistical analysis

GraphPad Prism 9 (GraphPad, San Diego, CA, USA; www.graphpad.com) and SPSS ver. 25 (IBM, Armonk, NY, USA) were used for the analysis. Data are expressed as the mean ± standard deviation (SD). To assess inter-group differences, the Student's t-test was used for *in vitro* experiments and the Mann-Whitney U nonparametric test was used for *in vivo* experiments (n=4 for each group). Statistical significance was set at P < 0.05, statistical details of experiments can be found in figure legends.

## Supplemental information

Data S1. Original data of Western blot, related to WB analysis and IP section in STAR methods

Document S1. Figures S1–S10

Table S1. Primers for qPCR, related to STAR method Real-time qPCR section

## References

[1] Agha O., Diaz A., Davies M., Kim H.T., Liu X., Feeley B.T. (2021). Rotator cuff tear degeneration and the role of fibro-adipogenic progenitors. Ann. N. Y. Acad. Sci..

[2] Redler L.H., Dennis E.R. (2019). Treatment of Adhesive Capsulitis of the Shoulder. J. Am. Acad. Orthop. Surg..

[3] Valencia A.P., Lai J.K., Iyer S.R., Mistretta K.L., Spangenburg E.E., Davis D.L., Lovering R.M., Gilotra M.N. (2018). Fatty Infiltration Is a Prognostic Marker of Muscle Function After Rotator Cuff Tear. Am. J. Sports Med..

[4] Longo U.G., Carnevale A., Piergentili I., Berton A., Candela V., Schena E., Denaro V. (2021). Retear rates after rotator cuff surgery: a systematic review and meta-analysis. BMC Musculoskelet. Disord..

[5] Zhao J., Luo M., Pan J., Liang G., Feng W., Zeng L., Yang W., Liu J. (2021). Risk factors affecting rotator cuff retear after arthroscopic repair: a meta-analysis and systematic review. J. Shoulder Elbow Surg..

[6] Bryniarski A.R., Meyer G.A. (2019). Brown Fat Promotes Muscle Growth During Regeneration. J. Orthop. Res..

[7] Wang Z., Liu X., Jiang K., Kim H., Kajimura S., Feeley B.T. (2020). Intramuscular Brown Fat Activation Decreases Muscle Atrophy and Fatty Infiltration and Improves Gait After Delayed Rotator Cuff Repair in Mice. Am. J. Sports Med..

[8] Liu X., Liu M., Lee L., Davies M., Wang Z., Kim H., Feeley B.T. (2021). Trichostatin A regulates fibro/adipogenic progenitor adipogenesis epigenetically and reduces rotator cuff muscle fatty infiltration. J. Orthop. Res..

[9] Wang Z., Liu R., Chen L., Wang H., Zhou M., Wang Y., Qin Y. (2021). Pharmacokinetics of Ginsenoside Rh2, the Major Anticancer Ingredient of Ginsenoside H Dripping Pills, in Healthy Subjects. Clin. Pharmacol. Drug Dev..

[10] Lee T.X.Y., Wu J., Jean W.H., Condello G., Alkhatib A., Hsieh C.C., Hsieh Y.W., Huang C.Y., Kuo C.H. (2021). Reduced stem cell aging in exercised human skeletal muscle is enhanced by ginsenoside Rg1. Aging.

[11] Fan W., Huang Y., Zheng H., Li S., Li Z., Yuan L., Cheng X., He C., Sun J. (2020). Ginsenosides for the treatment of metabolic syndrome and cardiovascular diseases: Pharmacology and mechanisms. Biomed. Pharmacother..

[12] Park S.J., Park M., Sharma A., Kim K., Lee H.J. (2019). Black Ginseng and Ginsenoside Rb1 Promote Browning by Inducing UCP1 Expression in 3T3-L1 and Primary White Adipocytes. Nutrients.

[13] Yang J.W., Kim S.S. (2015). Ginsenoside Rc promotes anti-adipogenic activity on 3T3-L1 adipocytes by down-regulating C/EBPα and PPARγ. Molecules.

[14] Yao L., Han Z., Zhao G., Xiao Y., Zhou X., Dai R., Han M., Wang Z., Xin R., Wang S. (2020). Ginsenoside Rd Ameliorates High Fat Diet-Induced Obesity by Enhancing Adaptive Thermogenesis in a cAMP-Dependent Manner. Obesity.

[15] Liu H., Liu M., Jin Z., Yaqoob S., Zheng M., Cai D., Liu J., Guo S. (2019). Ginsenoside Rg2 inhibits adipogenesis in 3T3-L1 preadipocytes and suppresses obesity in high-fat-diet-induced obese mice through the AMPK pathway. Food Funct..

[16] Zhao W., Yang J., Kang Y., Hu K., Jiao M., Zhao B., Jiang Y., Liu C., Ding F., Yuan B. (2022). Animal Models of Rotator Cuff Injury and Repair: A Systematic Review. Tissue Eng. Part B Rev..

[17] Li Z., Ji G.E. (2018). Ginseng and obesity. J. Ginseng Res..

[18] Koenen M., Hill M.A., Cohen P., Sowers J.R. (2021). Obesity, Adipose Tissue and Vascular Dysfunction. Circ. Res..

[19] Martinez-Lopez N., Singh R. (2015). Autophagy and Lipid Droplets in the Liver. Annu. Rev. Nutr..

[20] Chouchani E.T., Kazak L., Spiegelman B.M. (2019). New Advances in Adaptive Thermogenesis: UCP1 and Beyond. Cell Metab..

[21] Biferali B., Proietti D., Mozzetta C., Madaro L. (2019). Fibro-Adipogenic Progenitors Cross-Talk in Skeletal Muscle: The Social Network. Front. Physiol..

[22] Arrighi N., Moratal C., Clément N., Giorgetti-Peraldi S., Peraldi P., Loubat A., Kurzenne J.Y., Dani C., Chopard A., Dechesne C.A. (2015). Characterization of adipocytes derived from fibro/adipogenic progenitors resident in human skeletal muscle. Cell Death Dis..

[23] Molina T., Fabre P., Dumont N.A. (2021). Fibro-adipogenic progenitors in skeletal muscle homeostasis, regeneration and diseases. Open Biol..

[24] Xu Z., You W., Chen W., Zhou Y., Nong Q., Valencak T.G., Wang Y., Shan T. (2021). Single-cell RNA sequencing and lipidomics reveal cell and lipid dynamics of fat infiltration in skeletal muscle. J. Cachexia Sarcopenia Muscle.

[25] Morigny P., Boucher J., Arner P., Langin D. (2021). Lipid and glucose metabolism in white adipocytes: pathways, dysfunction and therapeutics. Nat. Rev. Endocrinol..

[26] Softic S., Meyer J.G., Wang G.X., Gupta M.K., Batista T.M., Lauritzen H.P.M.M., Fujisaka S., Serra D., Herrero L., Willoughby J. (2019). Dietary Sugars Alter Hepatic Fatty Acid Oxidation via Transcriptional and Post-translational Modifications of Mitochondrial Proteins. Cell Metab..

[27] Naiman S., Huynh F.K., Gil R., Glick Y., Shahar Y., Touitou N., Nahum L., Avivi M.Y., Roichman A., Kanfi Y. (2019). SIRT6 Promotes Hepatic Beta-Oxidation via Activation of PPARα. Cell Rep..

[28] Alrob O.A., Sankaralingam S., Ma C., Wagg C.S., Fillmore N., Jaswal J.S., Sack M.N., Lehner R., Gupta M.P., Michelakis E.D. (2014). Obesity-induced lysine acetylation increases cardiac fatty acid oxidation and impairs insulin signalling. Cardiovasc. Res..

[29] Houten S.M., Violante S., Ventura F.V., Wanders R.J.A. (2016). The Biochemistry and Physiology of Mitochondrial Fatty Acid β-Oxidation and Its Genetic Disorders. Annu. Rev. Physiol..

[30] Lapner P.L.C., Jiang L., Zhang T., Athwal G.S. (2015). Rotator cuff fatty infiltration and atrophy are associated with functional outcomes in anatomic shoulder arthroplasty. Clin. Orthop. Relat. Res..

[31] Tashjian R.Z. (2012). Epidemiology, natural history, and indications for treatment of rotator cuff tears. Clin. Sports Med..

[32] Moverman M.A., Puzzitiello R.N., Menendez M.E., Pagani N.R., Hart P.A.J., Churchill R.W., Kirsch J.M., Jawa A. (2022). Rotator cuff fatty infiltration and muscle atrophy: relation to glenoid deformity in primary glenohumeral osteoarthritis. J. Shoulder Elbow Surg..

[33] Lim S., Park J., Um J.Y. (2019). Ginsenoside Rb1 Induces Beta 3 Adrenergic Receptor-Dependent Lipolysis and Thermogenesis in 3T3-L1 Adipocytes and db/db Mice. Front. Pharmacol..

[34] Shen L., Xiong Y., Wang D.Q.H., Howles P., Basford J.E., Wang J., Xiong Y.Q., Hui D.Y., Woods S.C., Liu M. (2013). Ginsenoside Rb1 reduces fatty liver by activating AMP-activated protein kinase in obese rats. J. Lipid Res..

[35] Huang Q., Su H., Qi B., Wang Y., Yan K., Wang X., Li X., Zhao D. (2021). A SIRT1 Activator, Ginsenoside Rc, Promotes Energy Metabolism in Cardiomyocytes and Neurons. J. Am. Chem. Soc..

[36] Rao J.N., Warren G.Z.L., Estolt-Povedano S., Zammit V.A., Ulmer T.S. (2011). An environment-dependent structural switch underlies the regulation of carnitine palmitoyltransferase 1A. J. Biol. Chem..

[37] Czuba L.C., Hillgren K.M., Swaan P.W. (2018). Post-translational modifications of transporters. Pharmacol. Ther..

[38] Liang K. (2023). Mitochondrial CPT1A: Insights into structure, function, and basis for drug development. Front. Pharmacol..

[39] Farup J., Just J., de Paoli F., Lin L., Jensen J.B., Billeskov T., Roman I.S., Cömert C., Møller A.B., Madaro L. (2021). Human skeletal muscle CD90+ fibro-adipogenic progenitors are associated with muscle degeneration in type 2 diabetic patients. Cell Metab..

[40] Dohmen R.G.J., Hubalek S., Melke J., Messmer T., Cantoni F., Mei A., Hueber R., Mitic R., Remmers D., Moutsatsou P. (2022). Muscle-derived fibro-adipogenic progenitor cells for production of cultured bovine adipose tissue. NPJ Sci. Food.

