# Supplementary material for: Ginsenoside Rb1 regulates CPT1A deacetylation to inhibit intramuscular fat infiltration after rotator cuff tear
Source: iScience. 2024 Jun 20;27(7):110331. doi: 10.1016/j.isci.2024.110331 (PMC11277379; doi:10.1016/j.isci.2024.110331)

iScience, Volume *27*

Supplemental information

Ginsenoside Rb1 regulates CPT1A deacetylation to inhibit intramuscular fat infiltration after rotator cuff tear

Yuesong Yin, Zili Wang, Yian Yang, Minren Shen, Hai Hu, Chuanshun Chen, Hecheng Zhou, Zheng Li, and Song Wu

Figure
4C-GAPDH

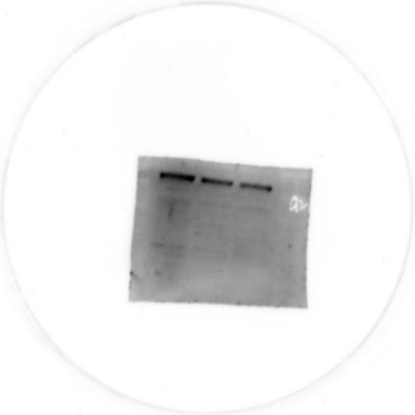

Figure 4C-Input ACADVL

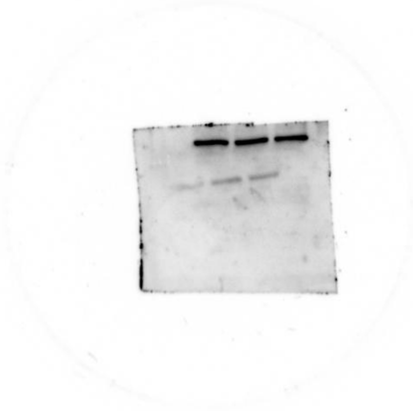

Figure 4C-IP ACADVL

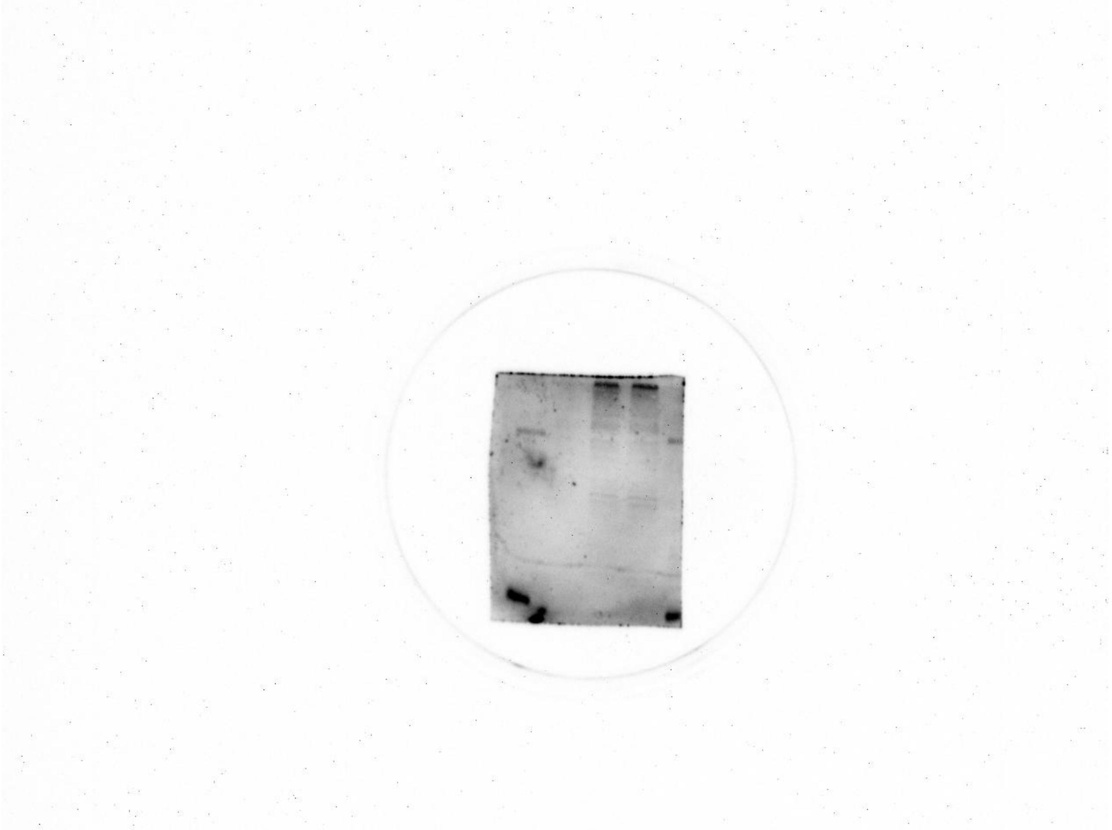

Figure 4C-PanAC

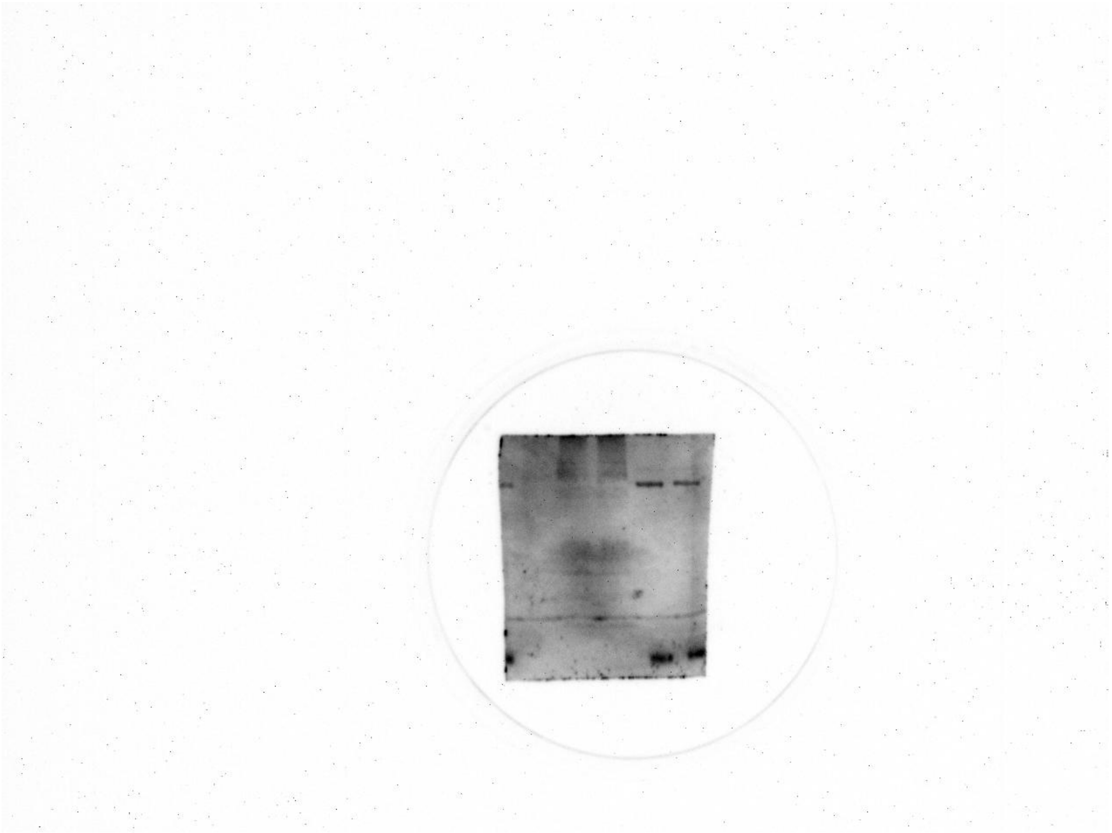

Figure 4D-GAPDH

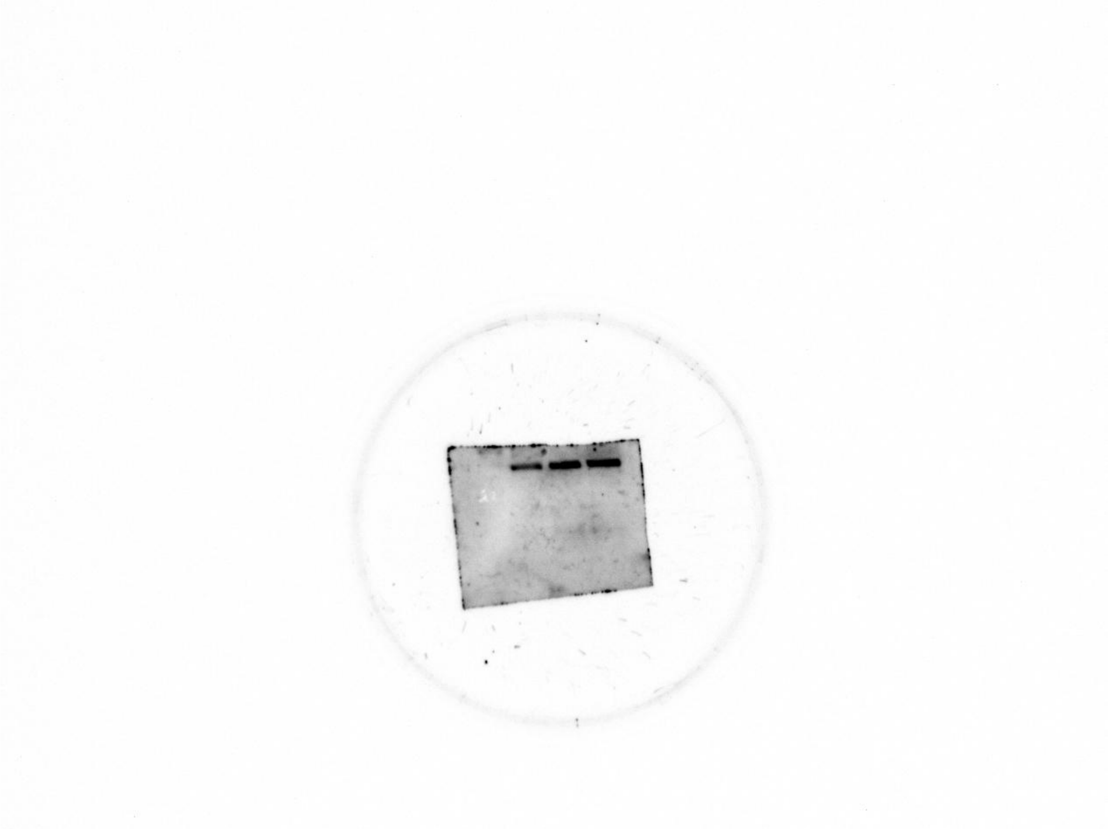

Figure 4D-Input ACADM

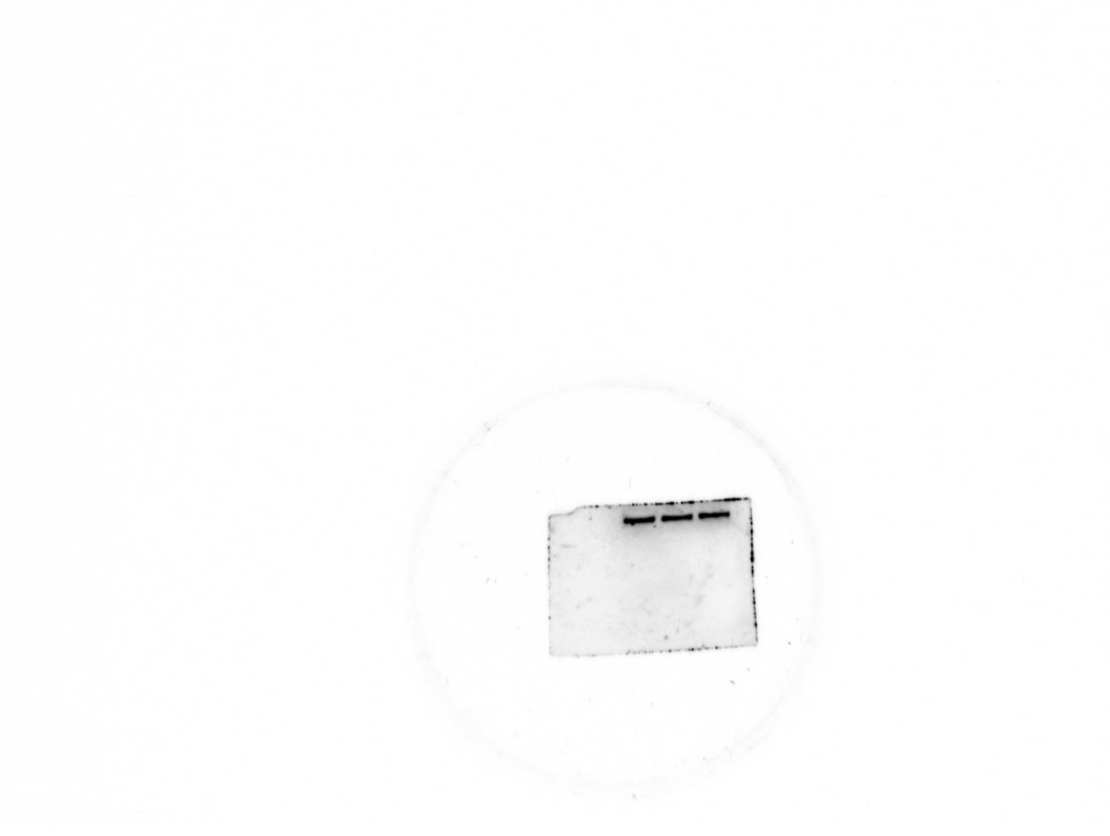

Figure 4D-IP ACADM

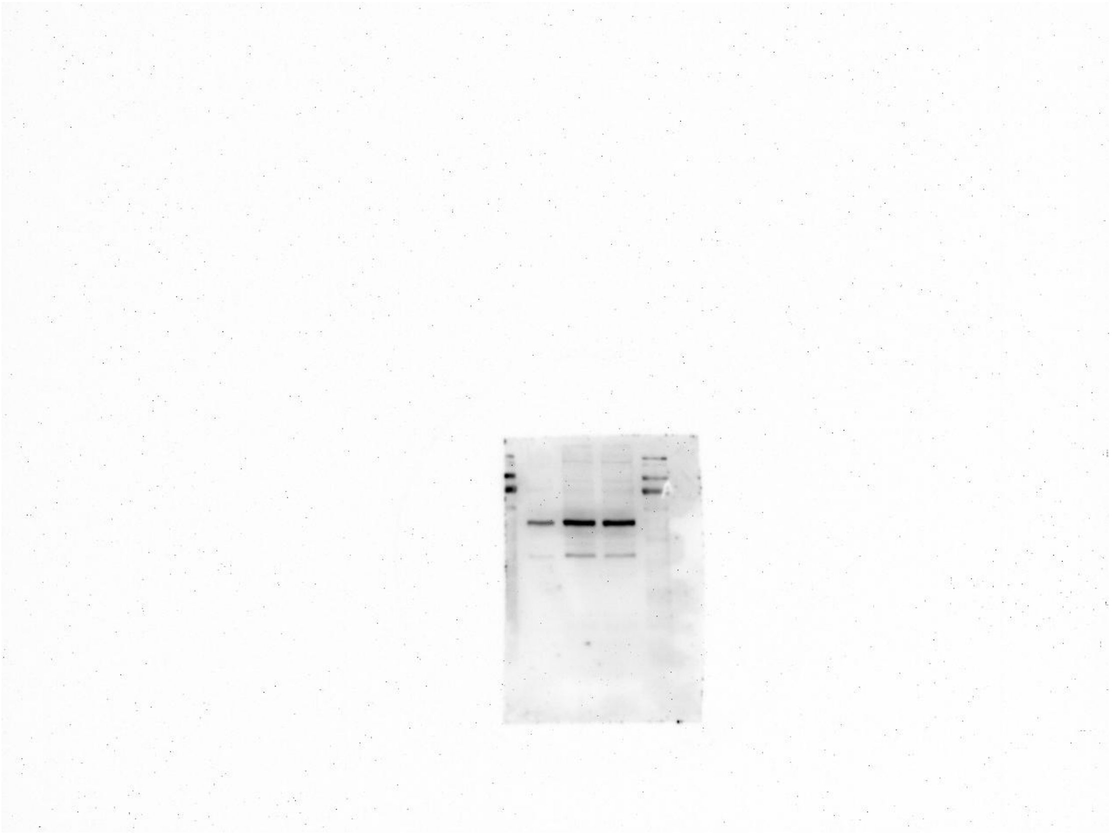

Figure 4D-PanAC

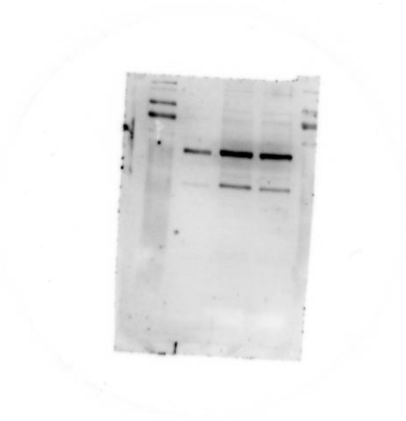

Figure 4E-GAPDH

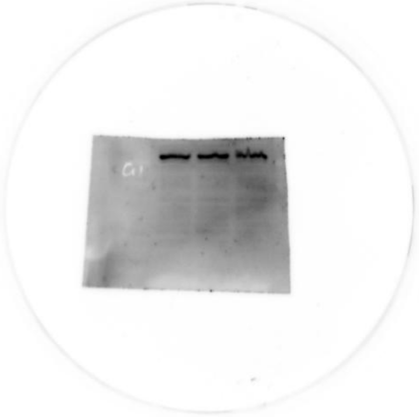

Figure 4E-Input ACADS

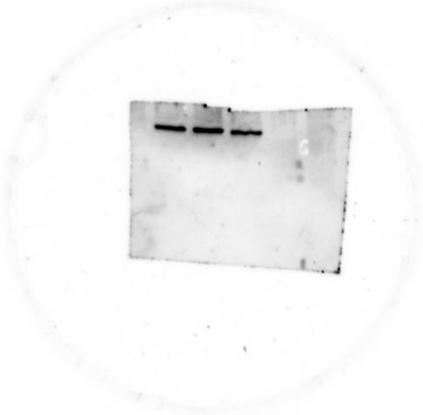

Figure 4E IP ACADS

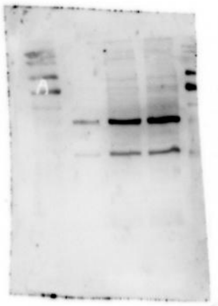

Figure 4E PanAC

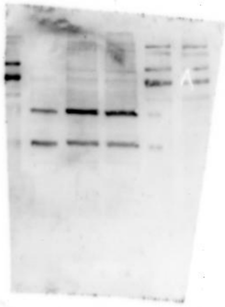

Figure 4F GAPDH

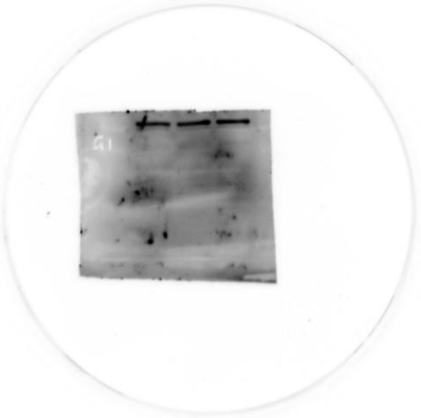

Figure 4F-Input ACAA2

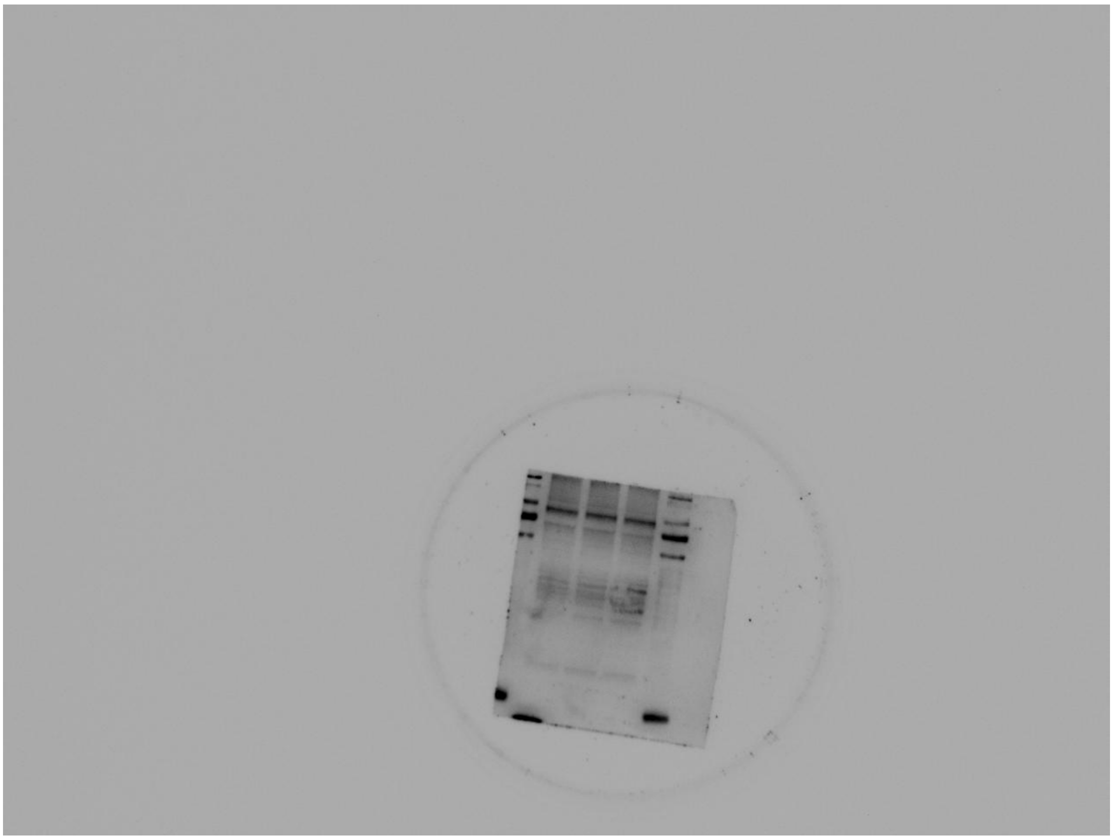

Figure 4F-IP ACAA2

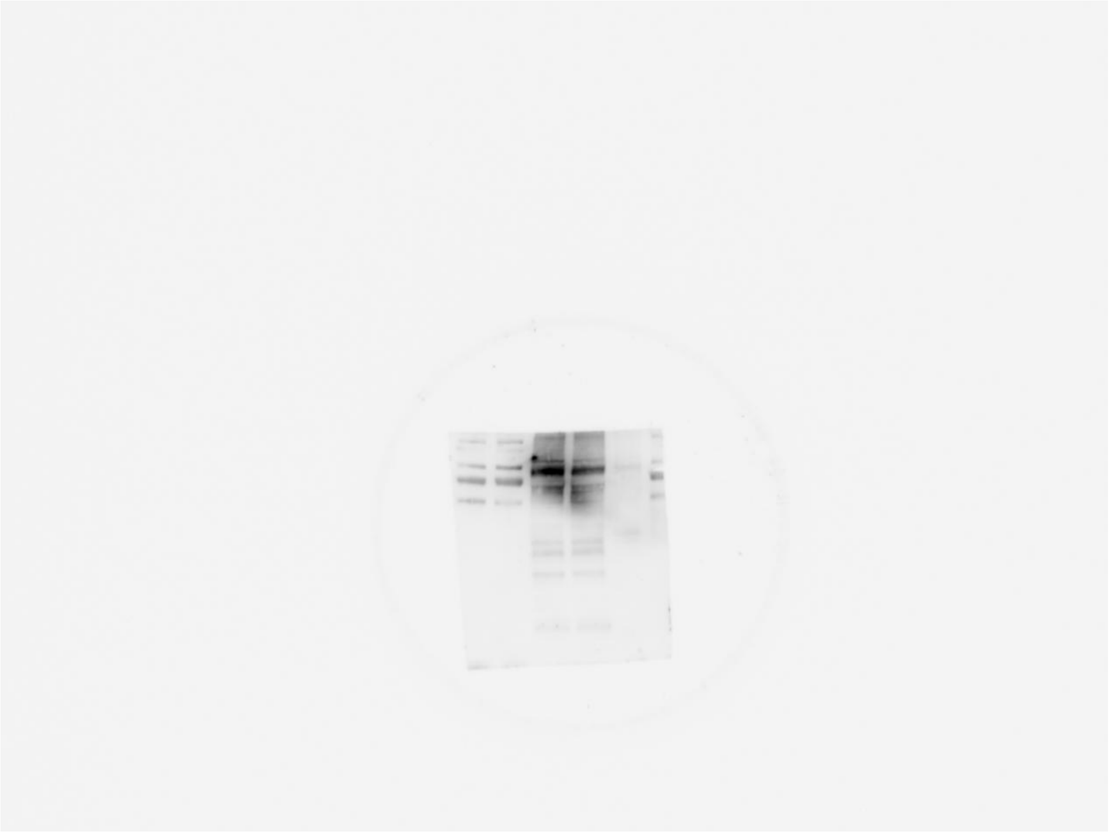

Figure 4F-PanAC

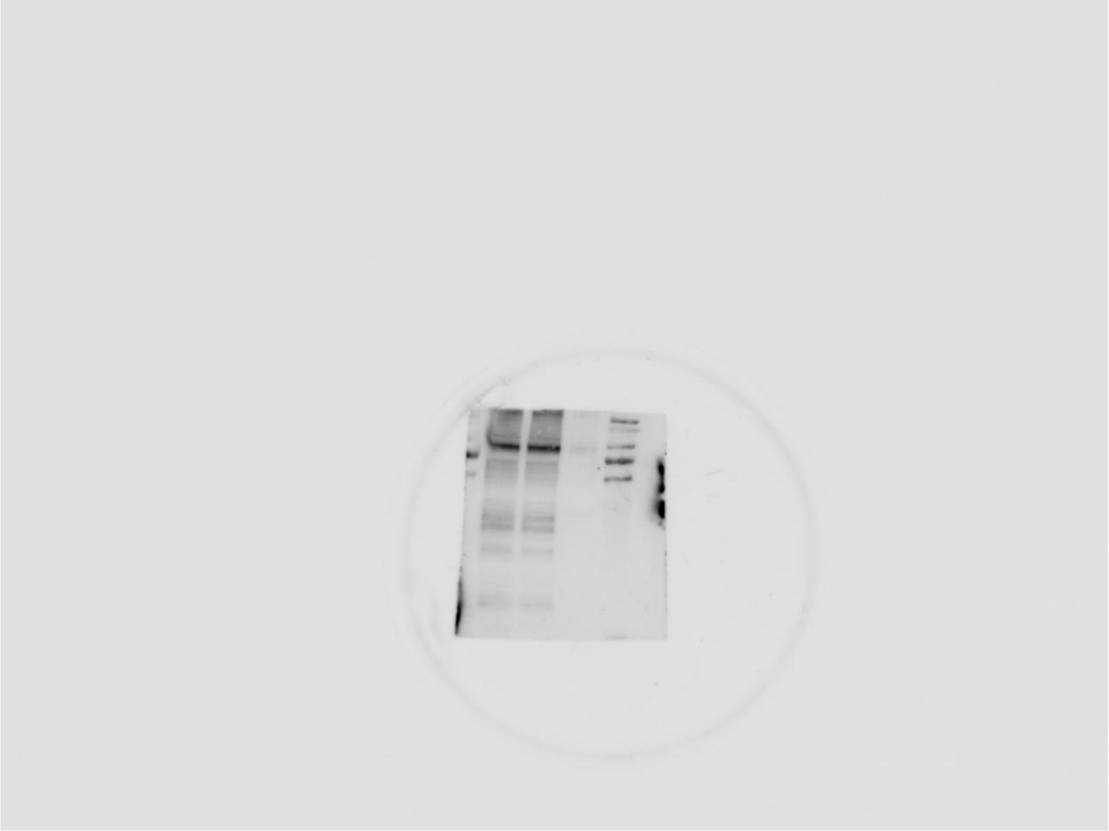

Figure 4G-GAPDH

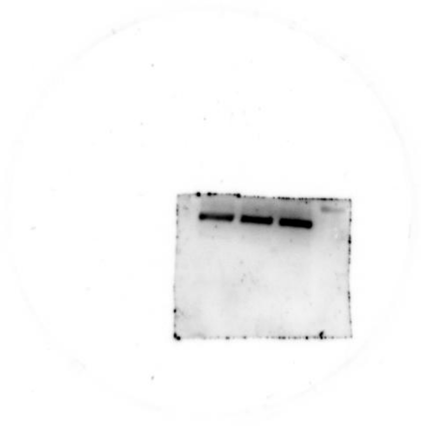

Figure 4G-Input CPT1A

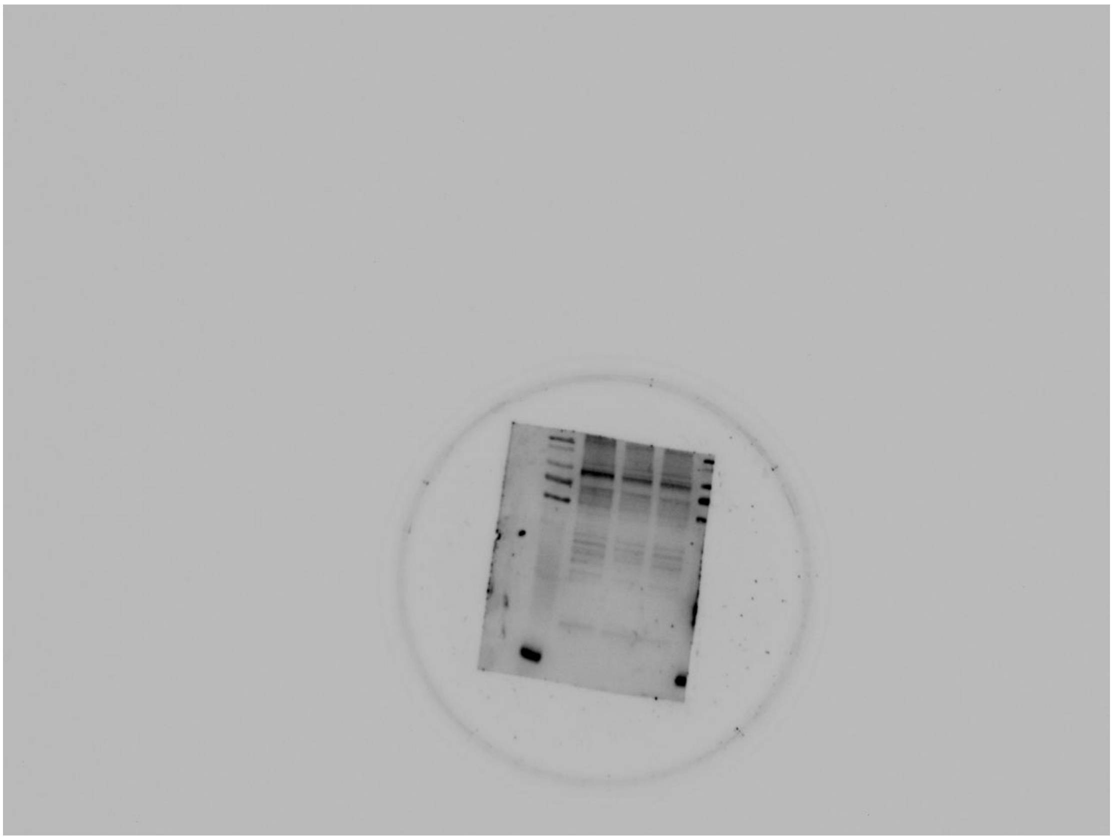

Figure 4G-IP CPT1A

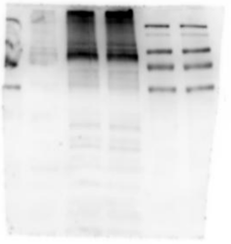

Figure 4G-PanAC

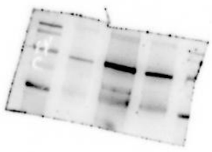

Figure 4H-GAPDH

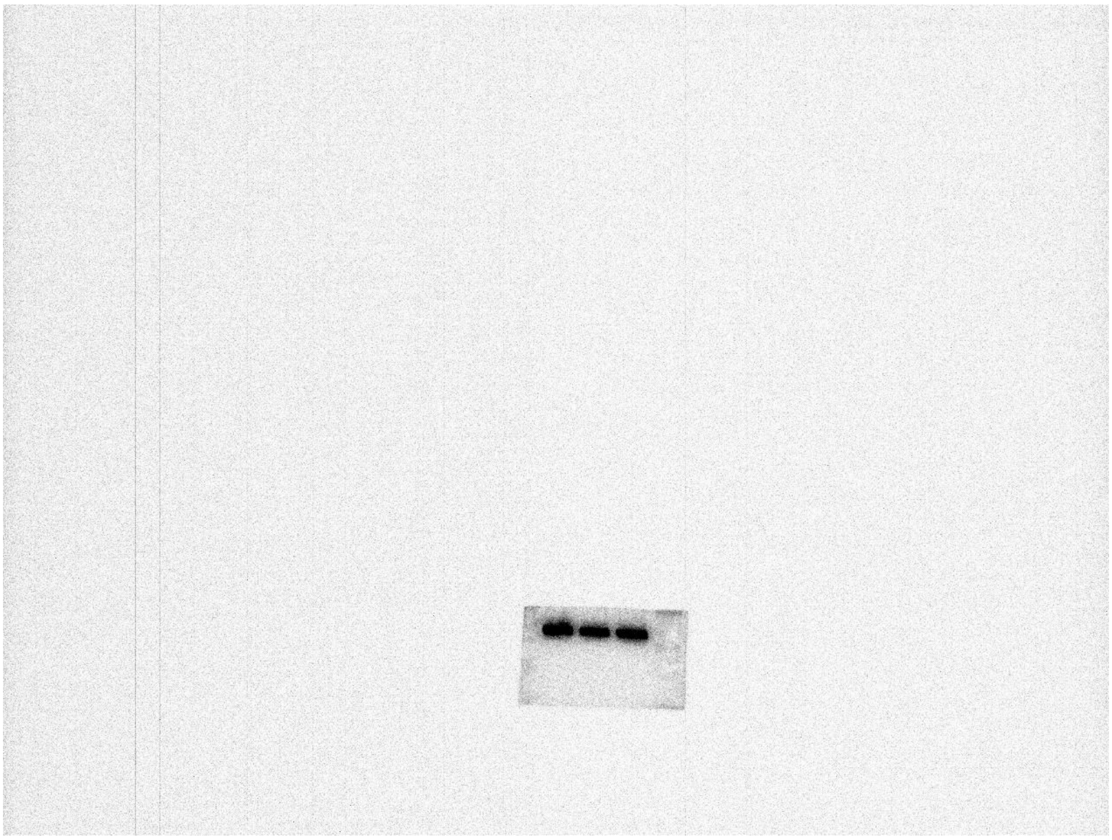

Figure 4H-Input ECHS1

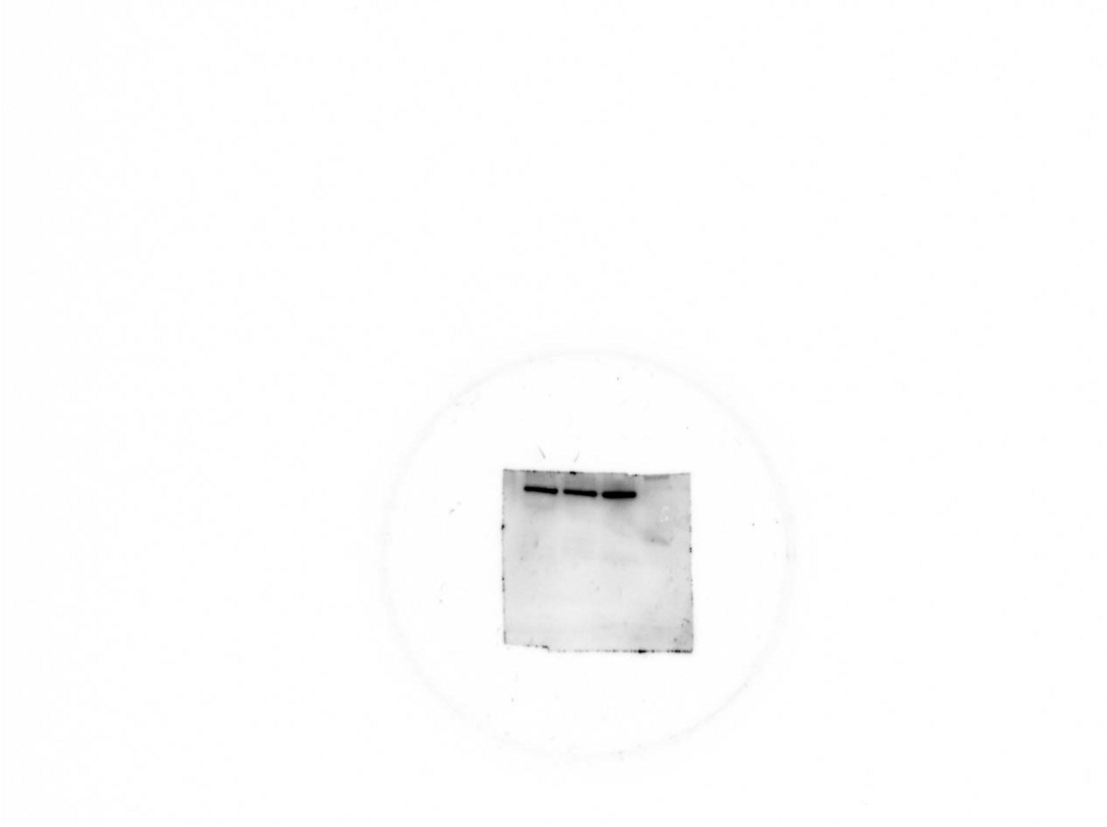

Figure 4H-IP ECHS1

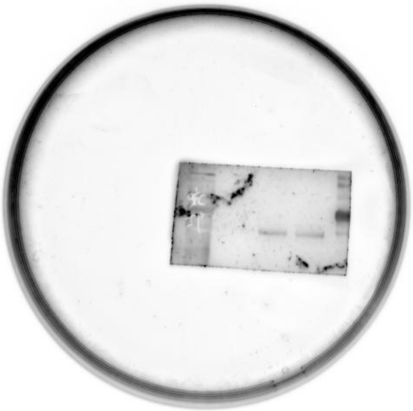

Figure 4H-PanAC

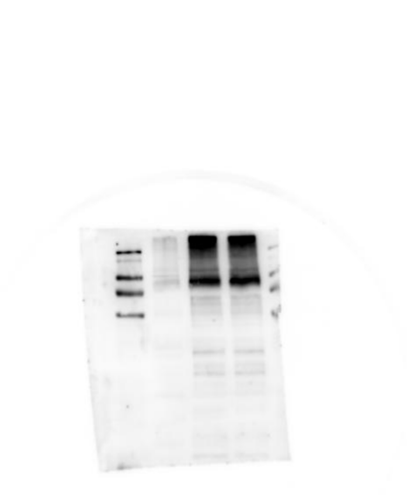

Figure 4I-GAPDH

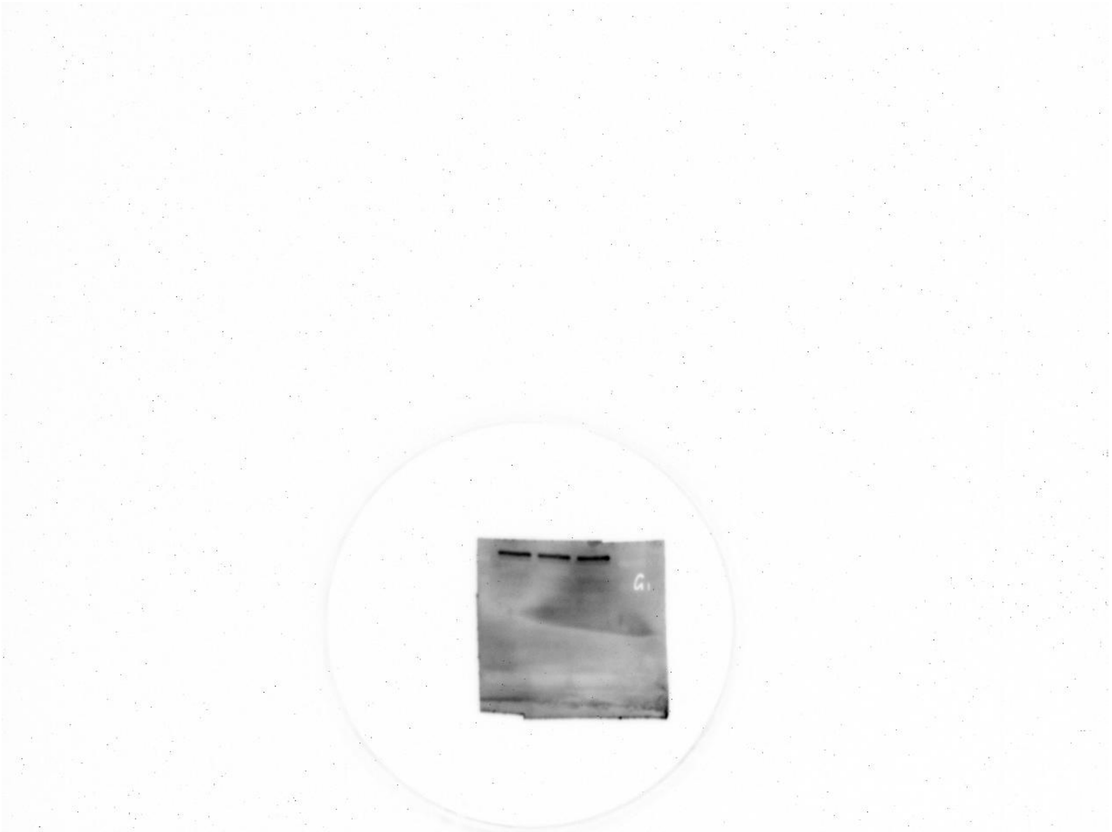

Figure 4I-INPUT CPT2

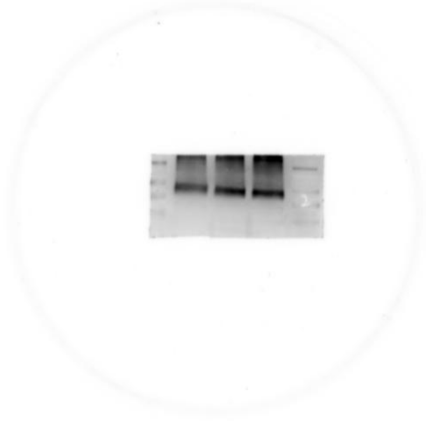

Figure 4I-IP CPT2

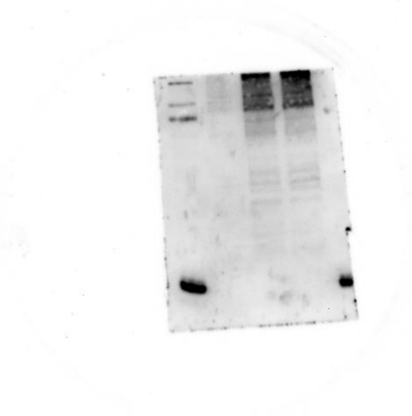

Figure 4I-PanAC

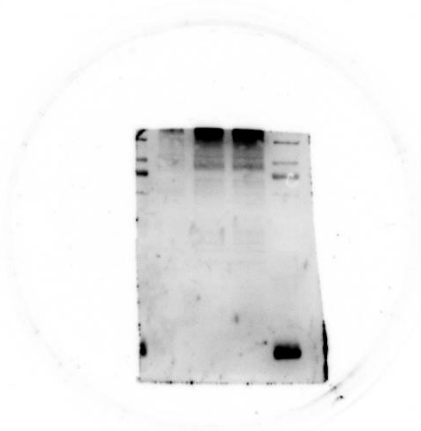

Figure 4J-GAPDH

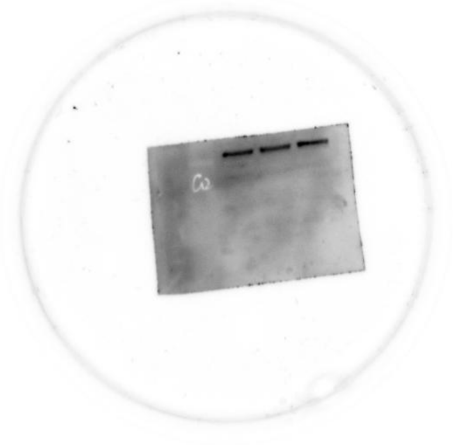

Figure 4J-INPUT HADH

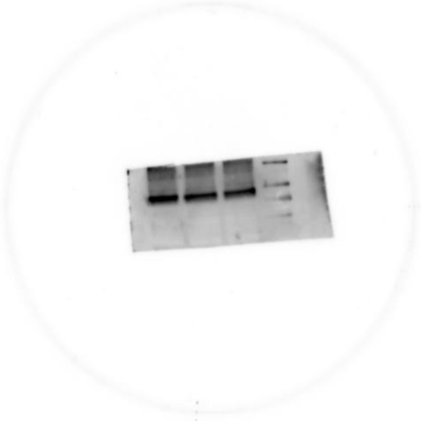

Figure 4J-IP HADH

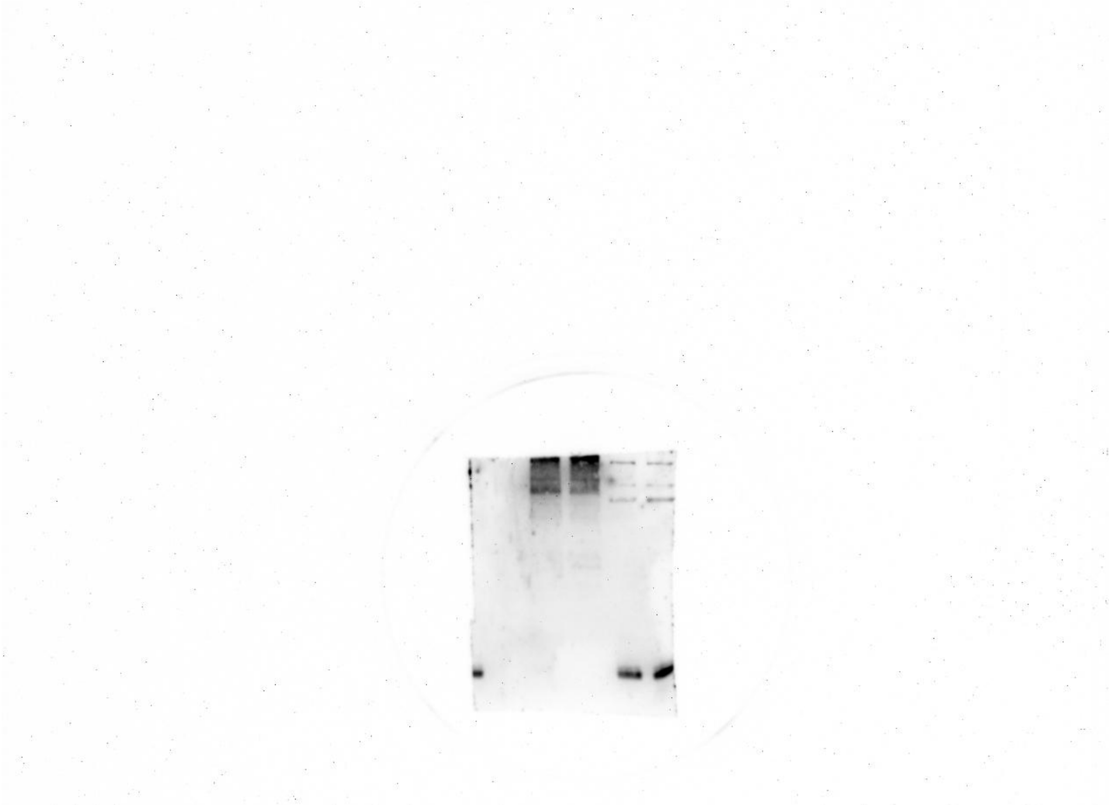

Figure 4J-PanAC

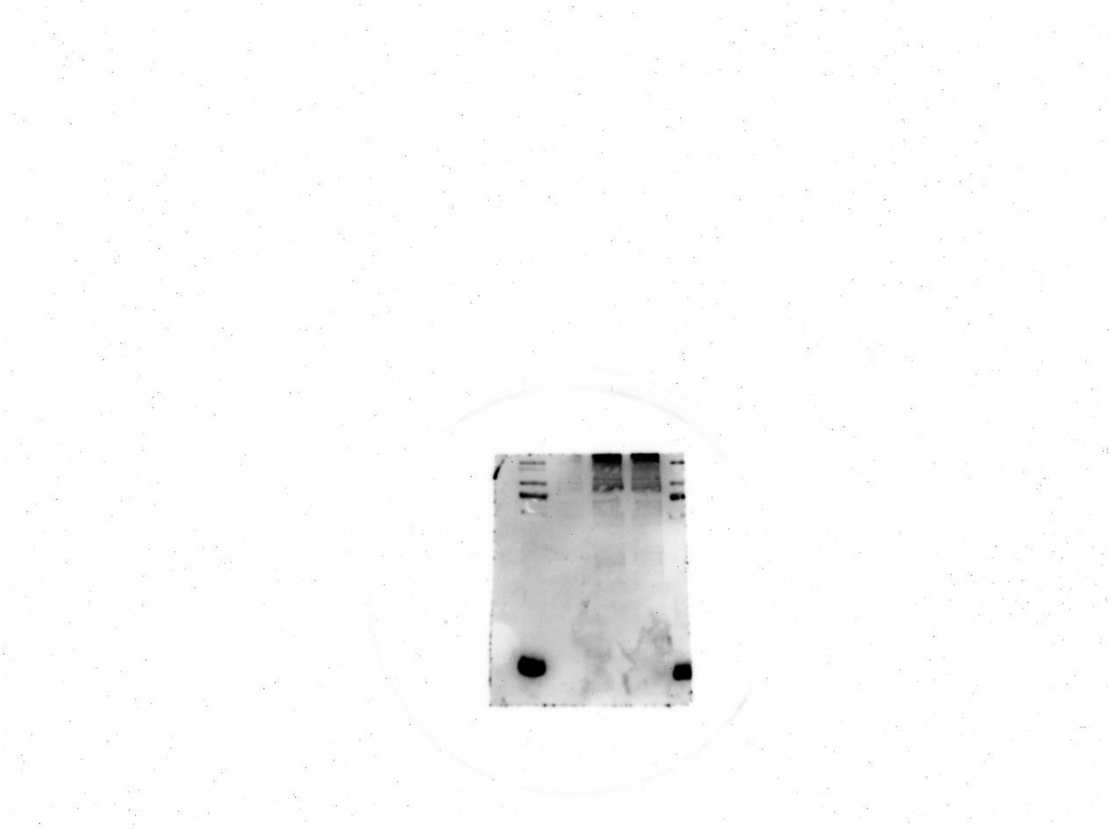

Figure 5B-IP CPT1A

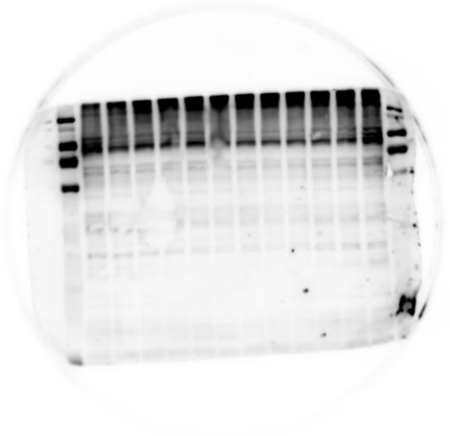

Figure 5B-FLAG

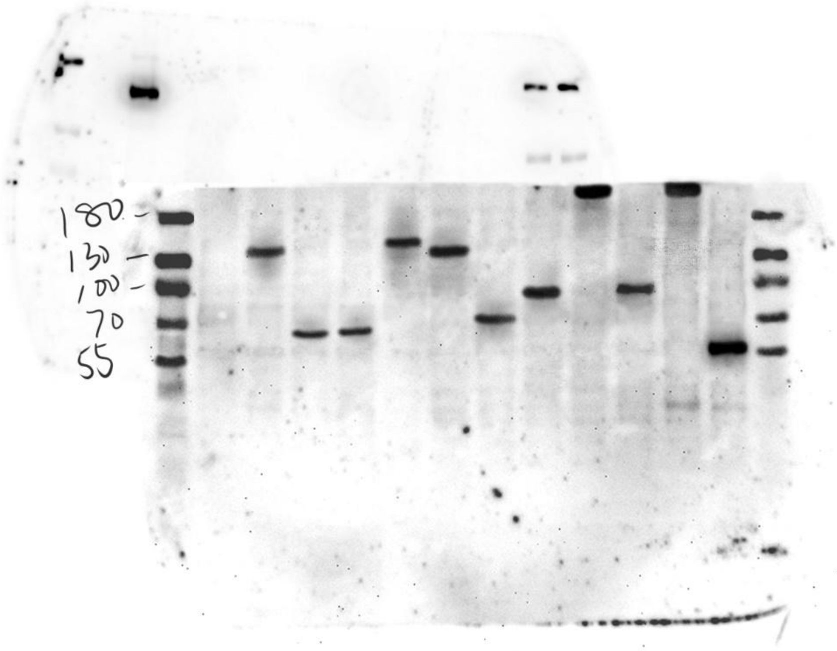

Figure 5B GAPDH

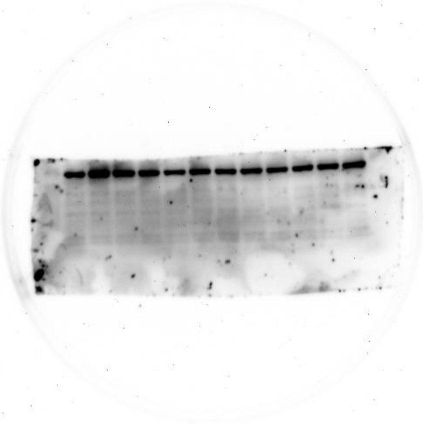

Figure 5B-Input CPT1A

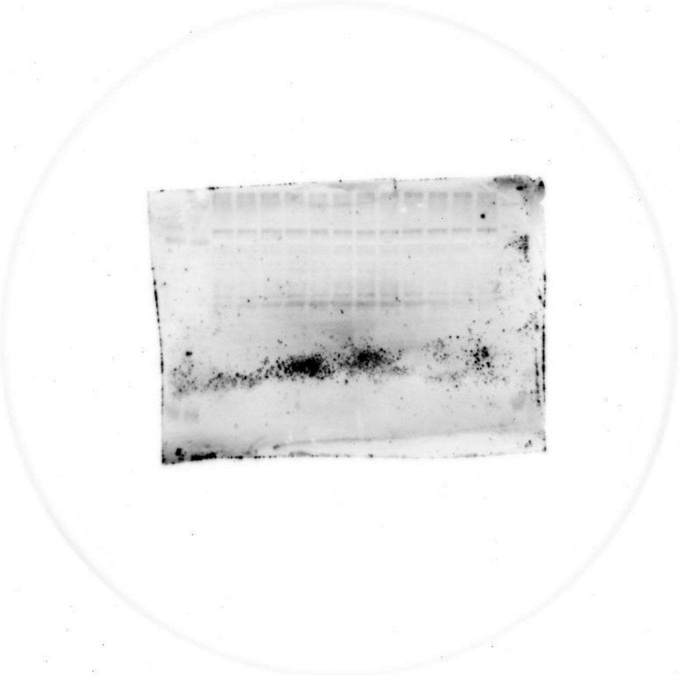

Figure 5B-PanAC

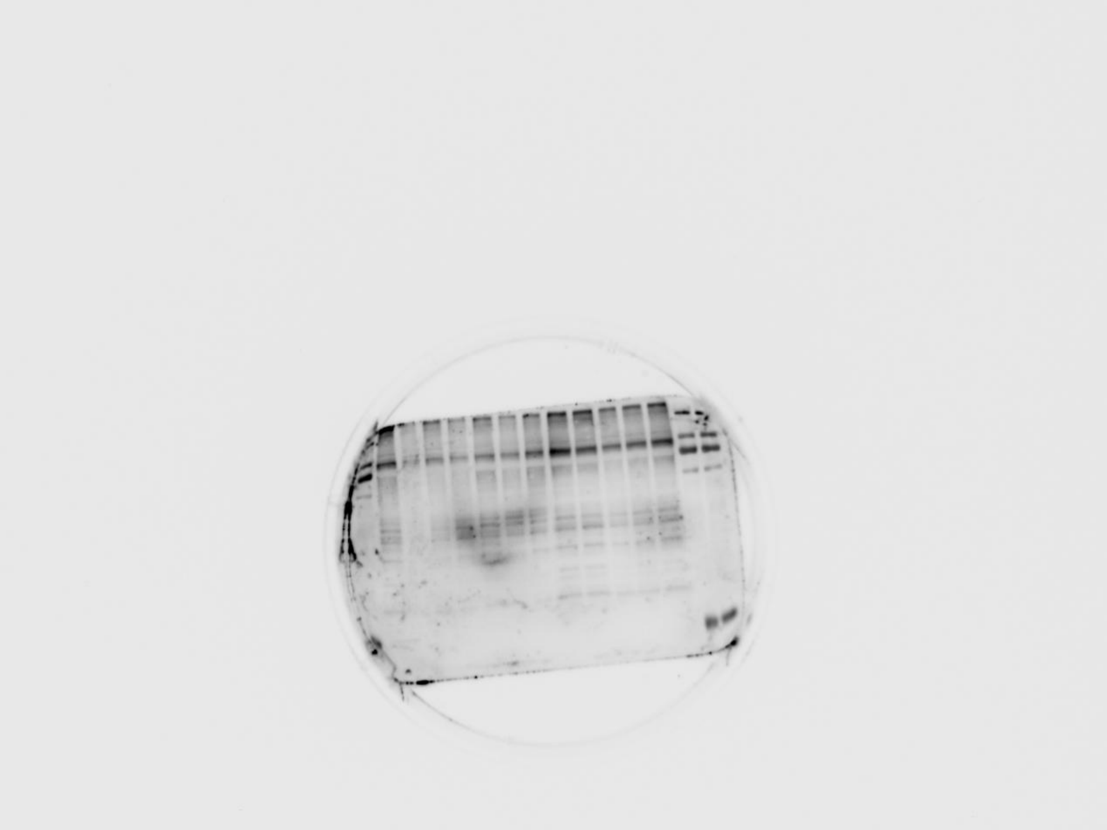

Figure 5D-GAPDH

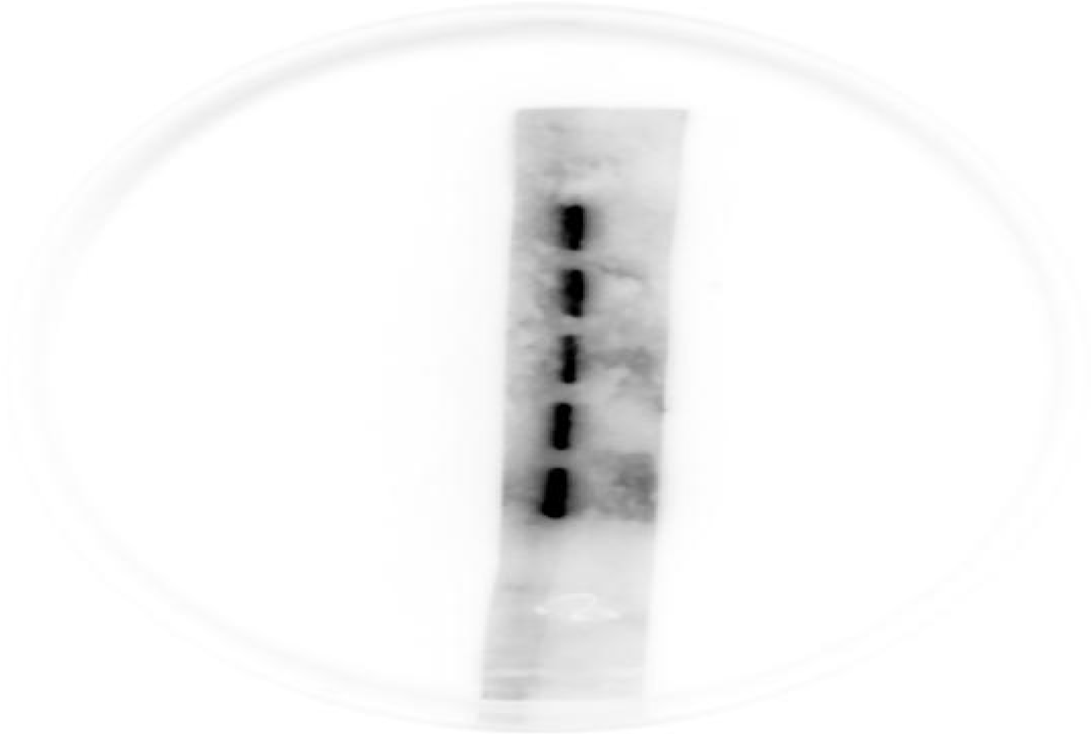

Figure 5D-INPUT CPT1A

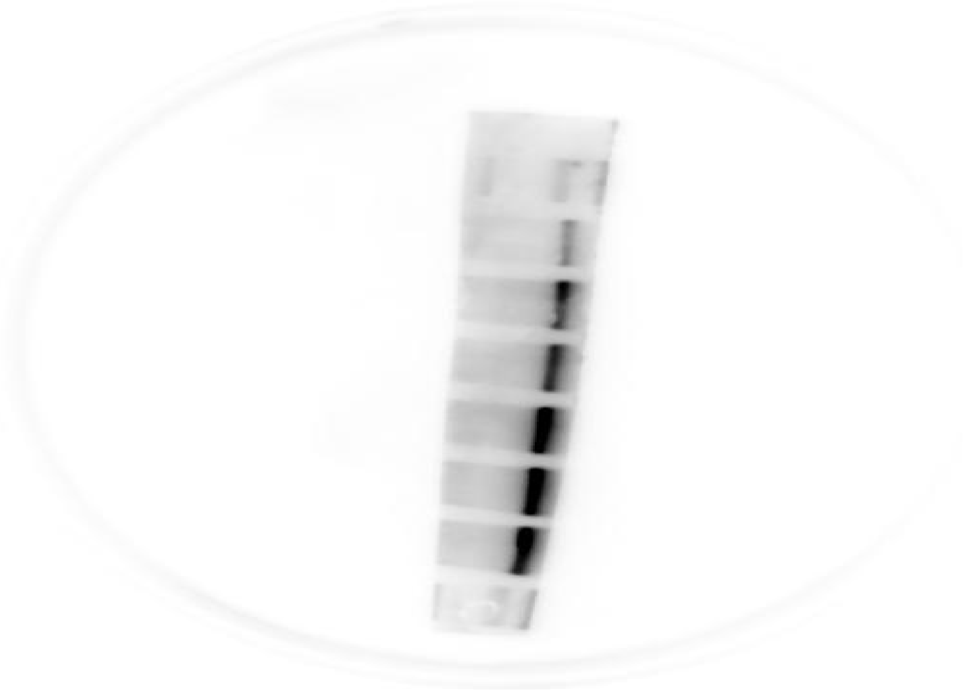

Figure 5D-INPUT SIRT1

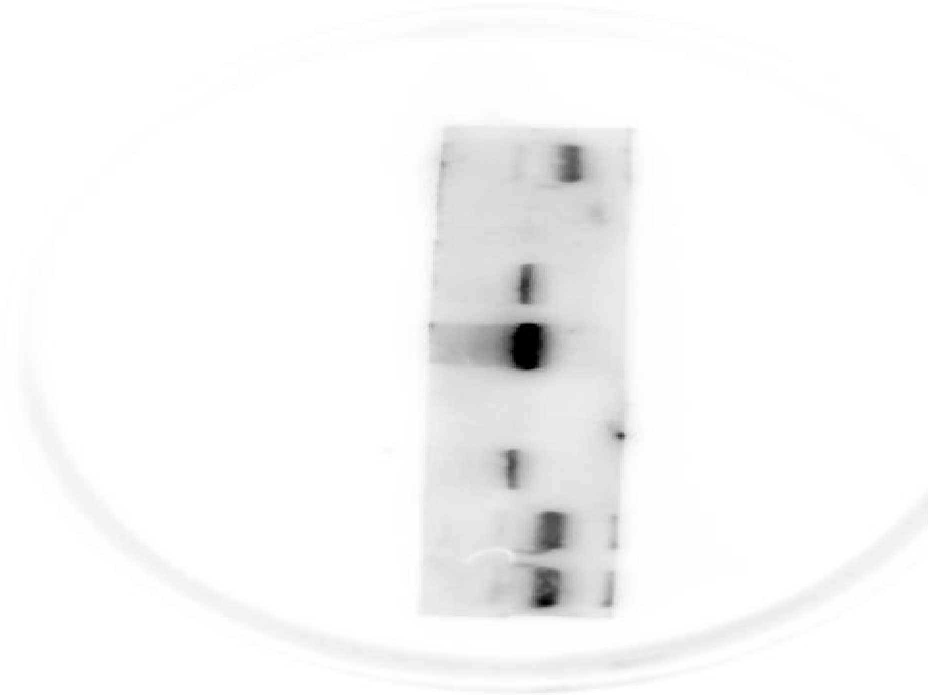

Figure 5D-IP CPT1A

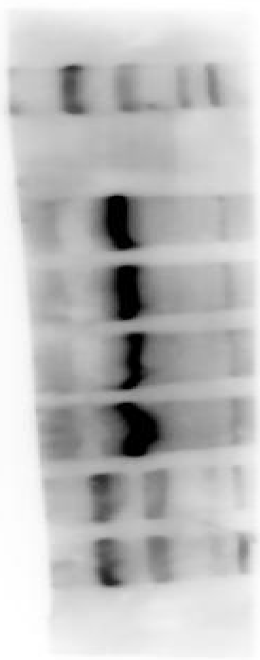

Figure 5D-PanAC

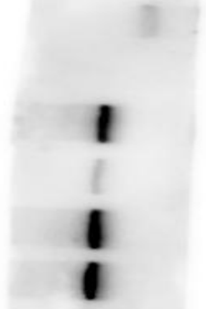

Figure 5E-CPT1A

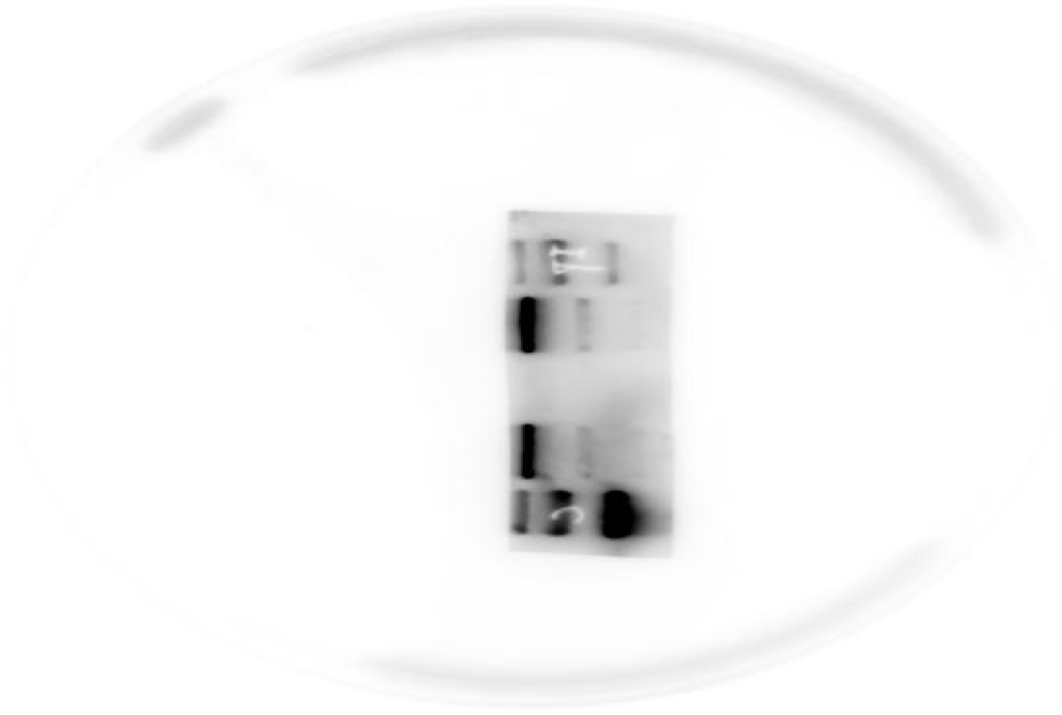

Figure 5E-FLAG SIRT1

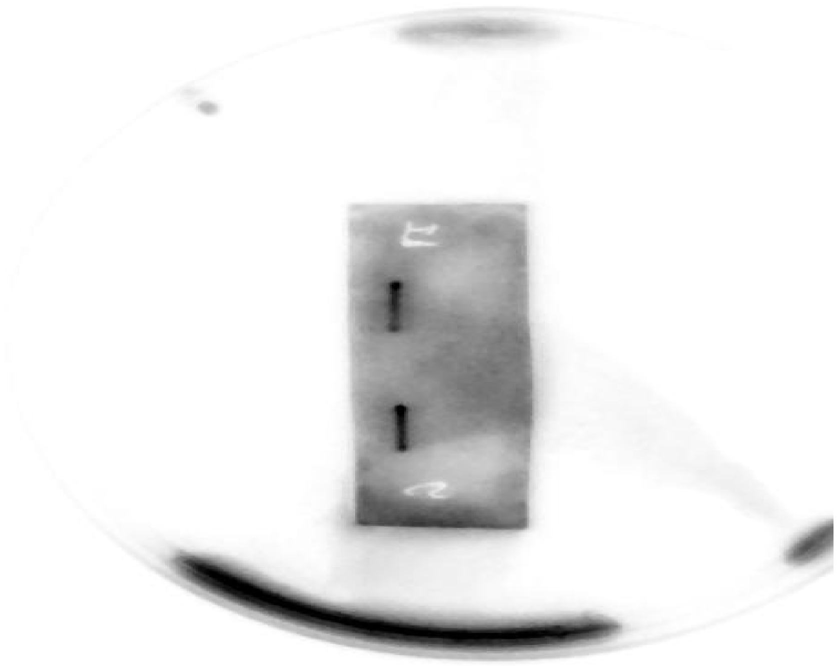

Figure 5F-FLAG CPT1A

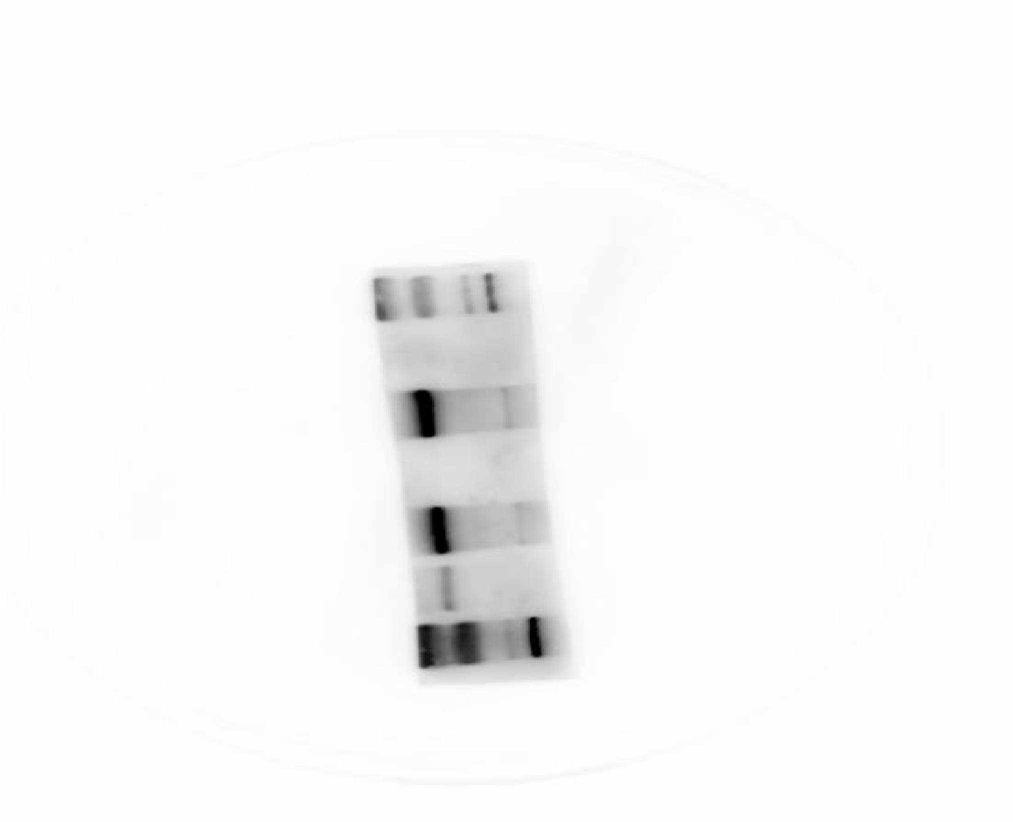

Figure 5F-SIRT1

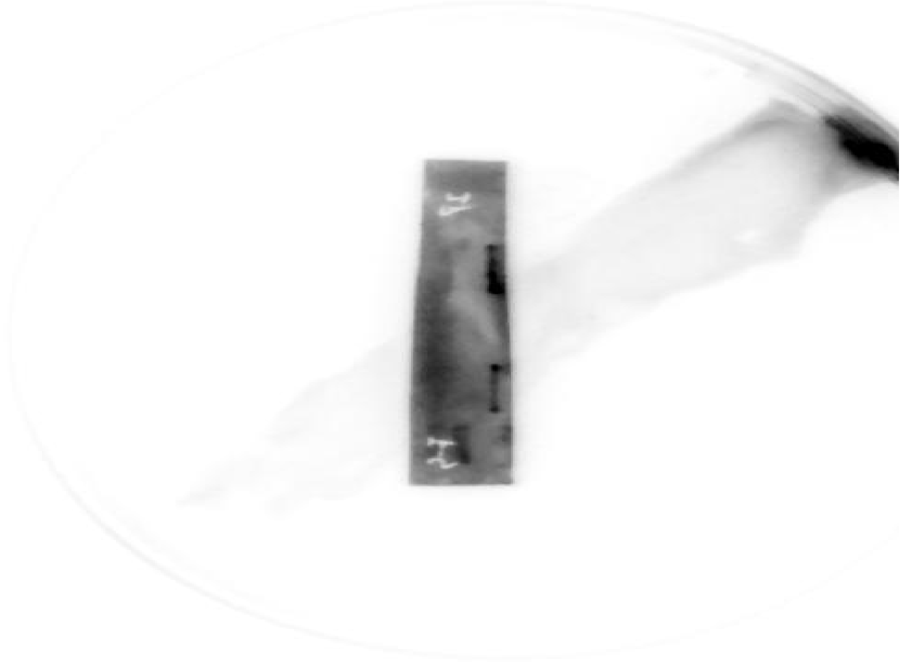

Figure 7B-Input CPT1A

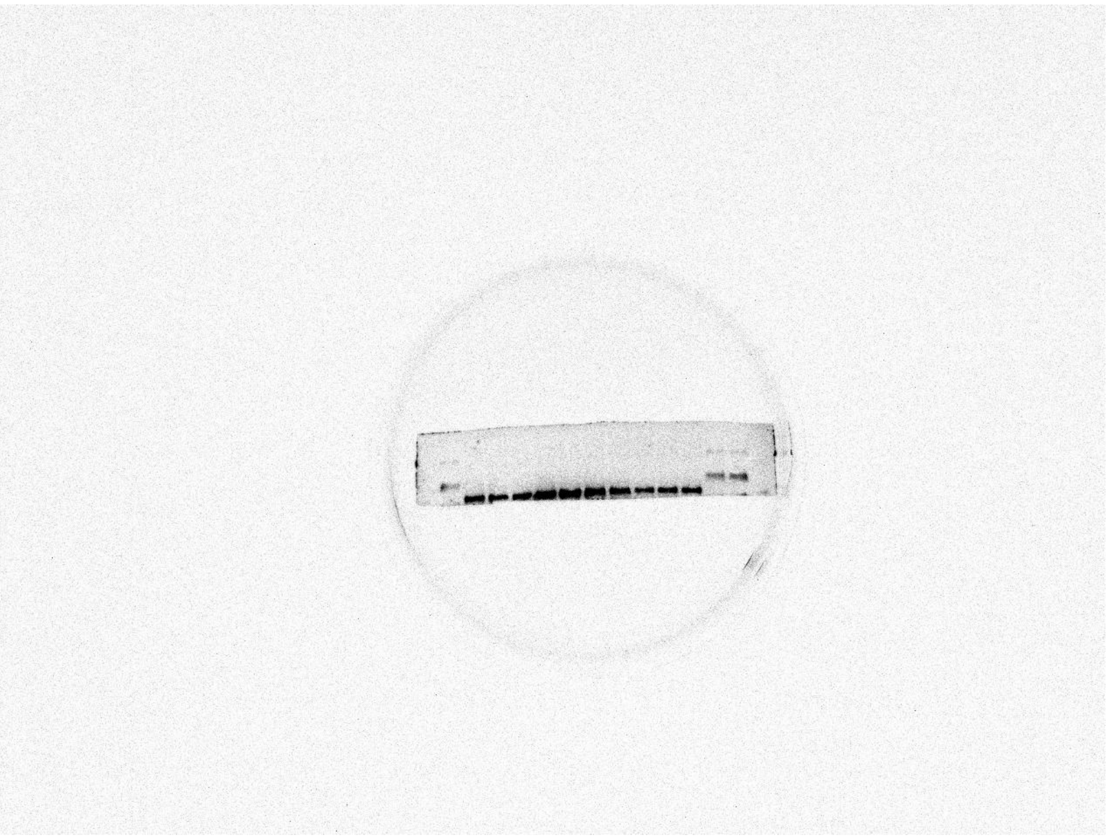

Figure 7B-IP CPT1A

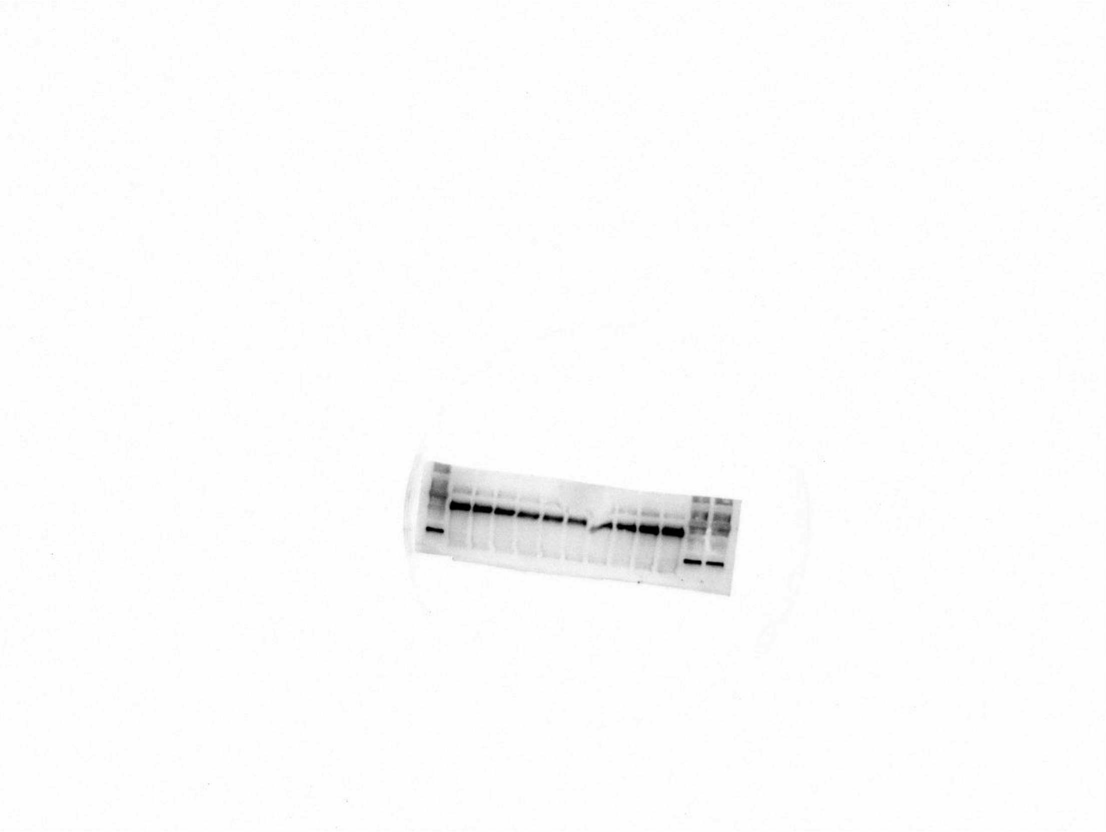

Figure 7B-GAPDH

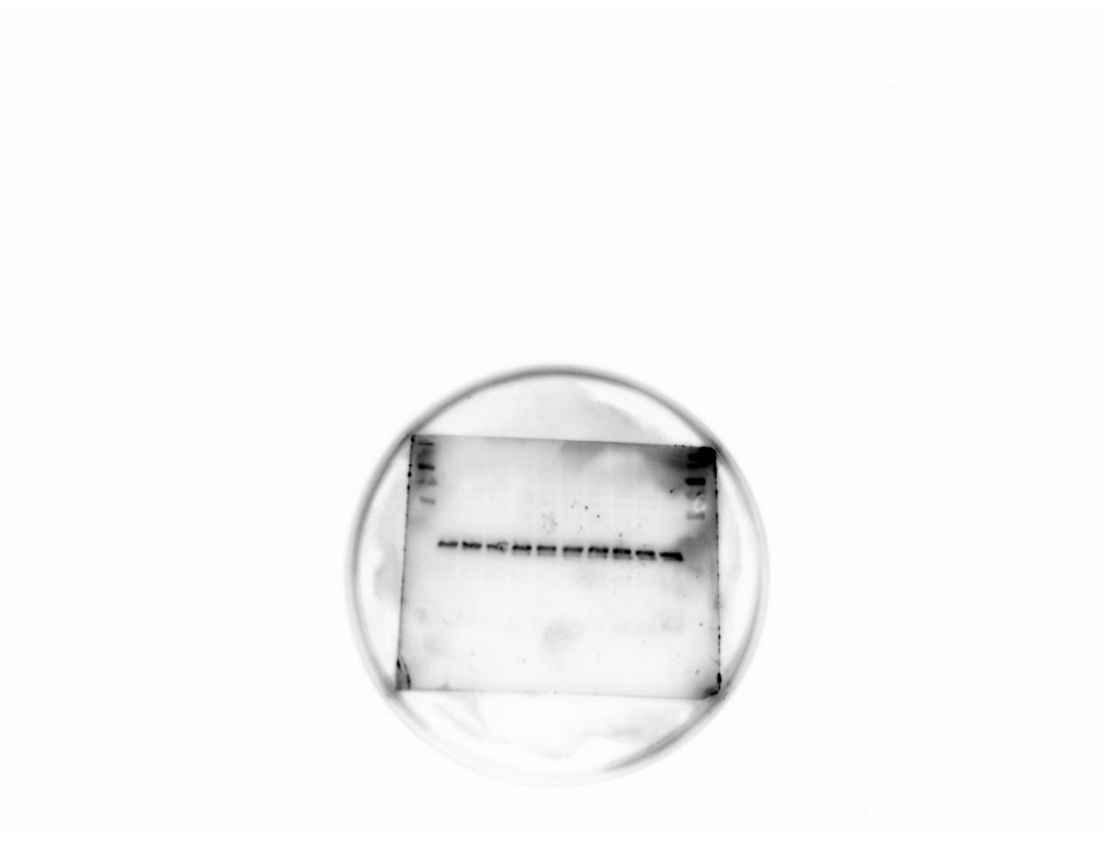

Figure 7B-INPUT SIRT1

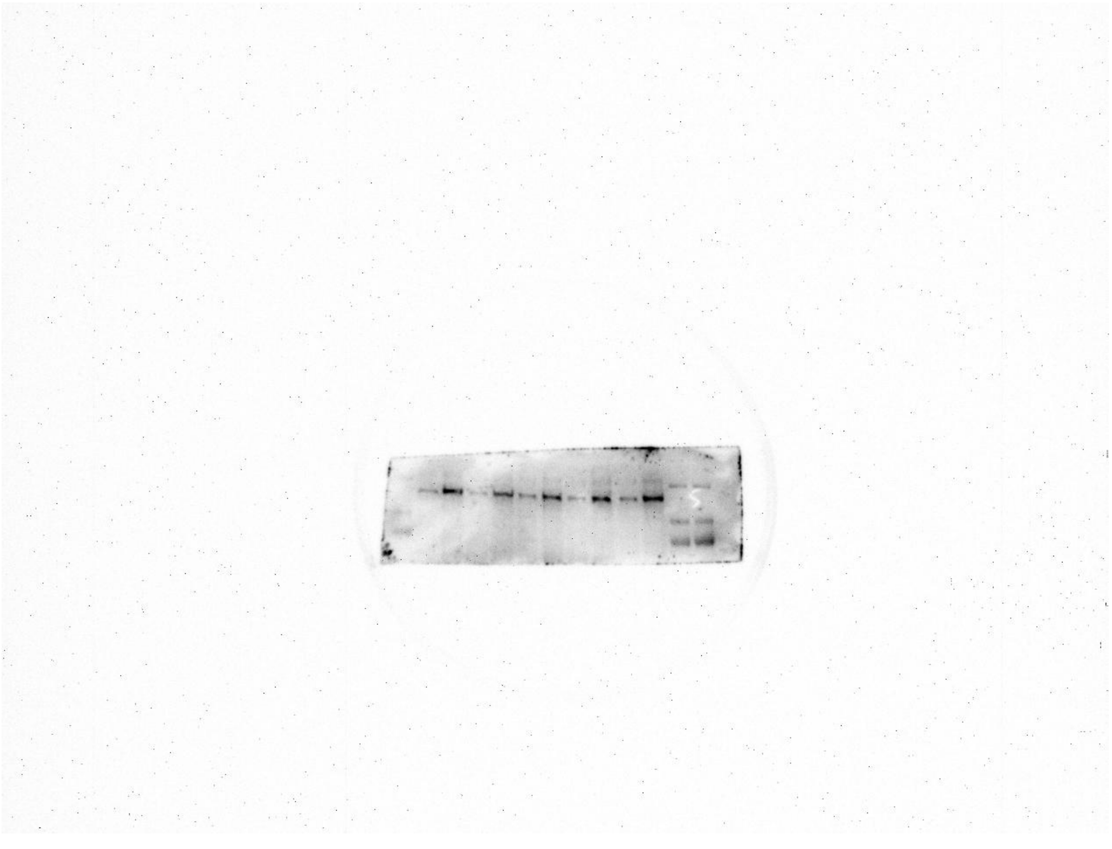

Figure 7B PanAC

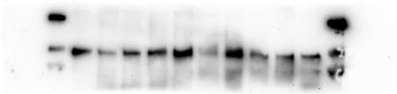

Figure 7C GAPDH

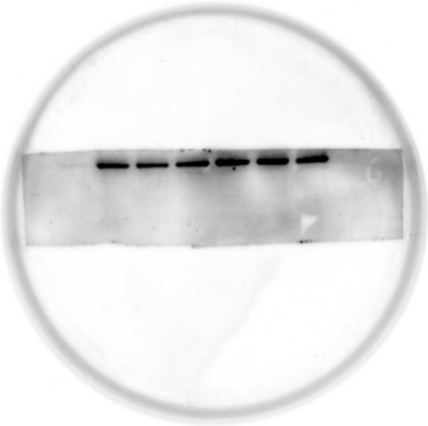

Figure 7C INPUT CPT1A

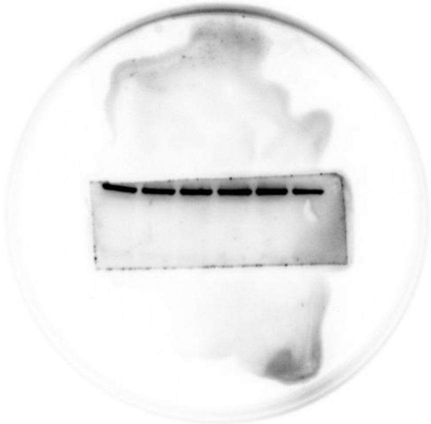

Figure 7C-INPUT SIRT1

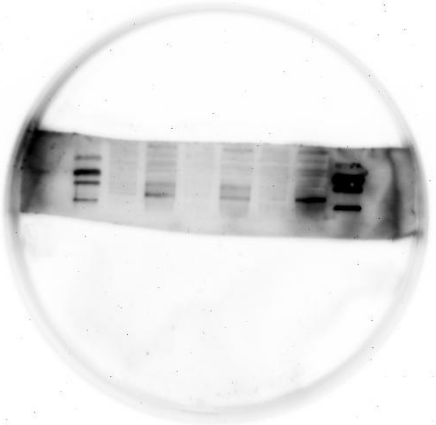

Figure 7C-IP CPT1A

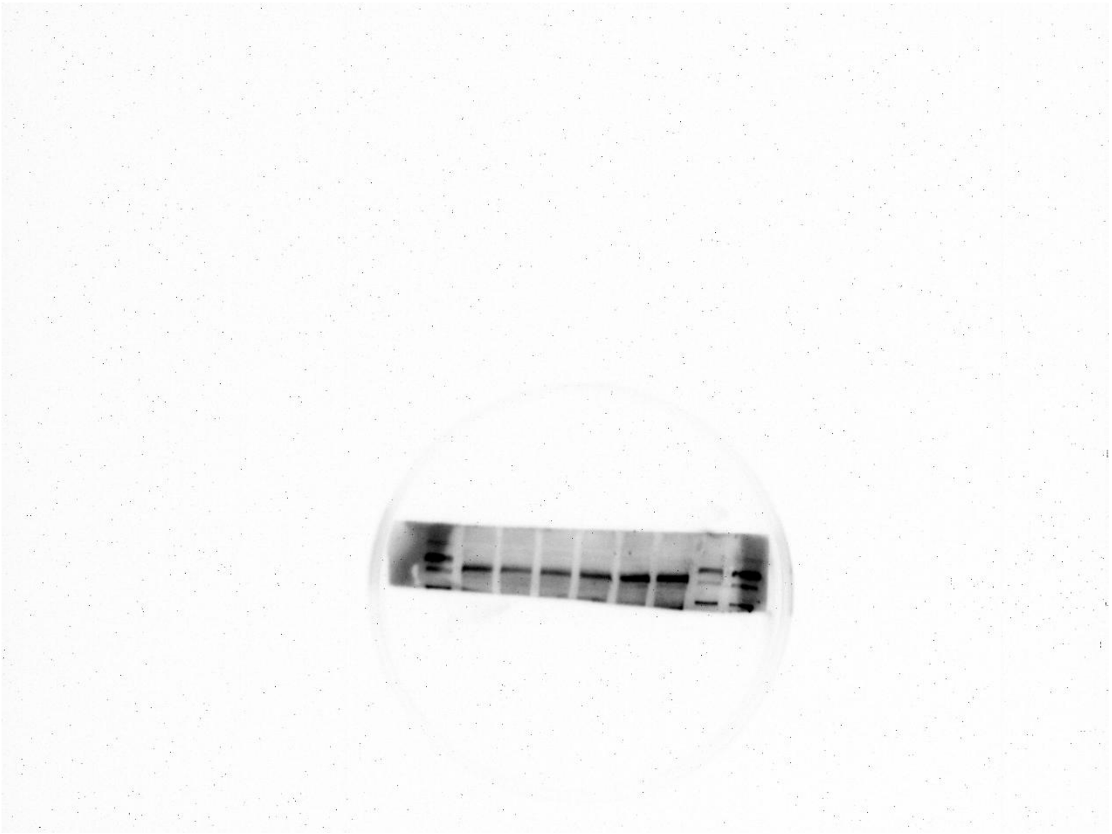

Figure 7C-PanAC

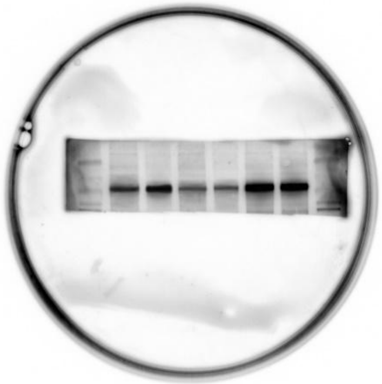

Figure S5A-GAPDH

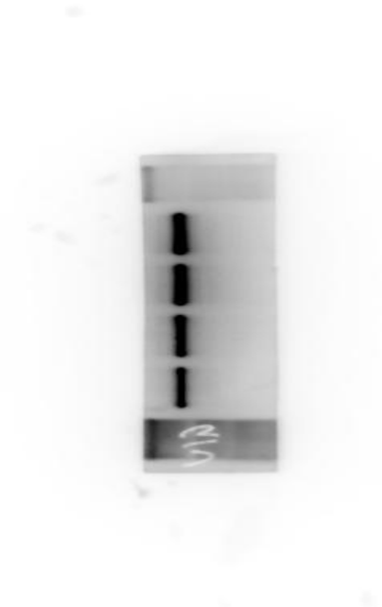

Figure S5A-SIRT1

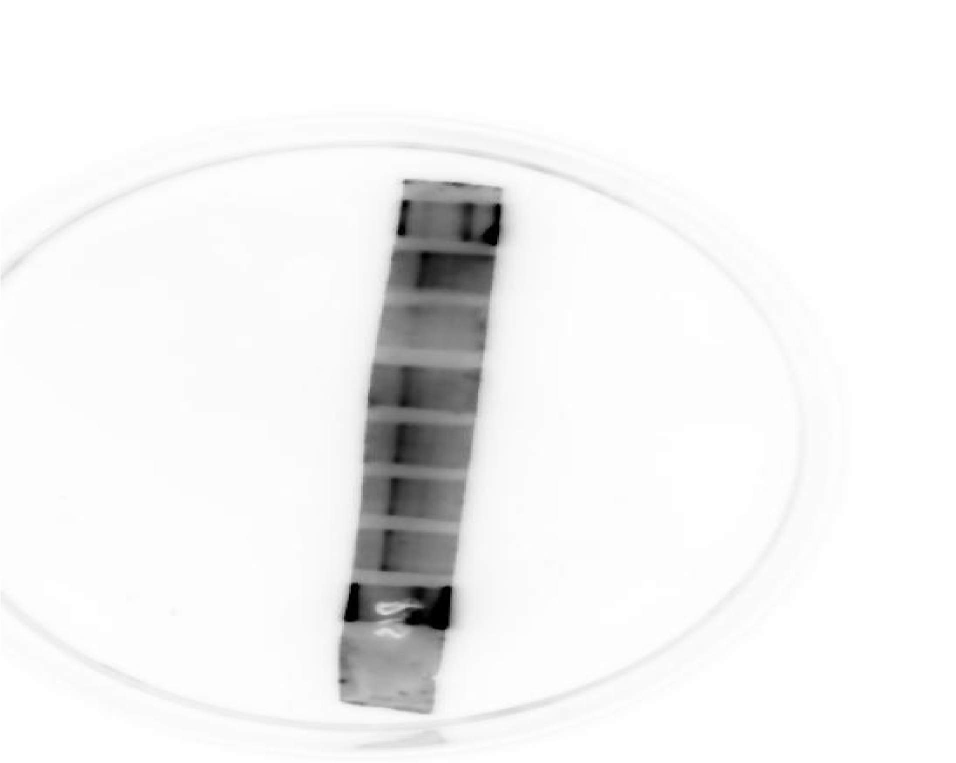

Figure S5C-GAPDH

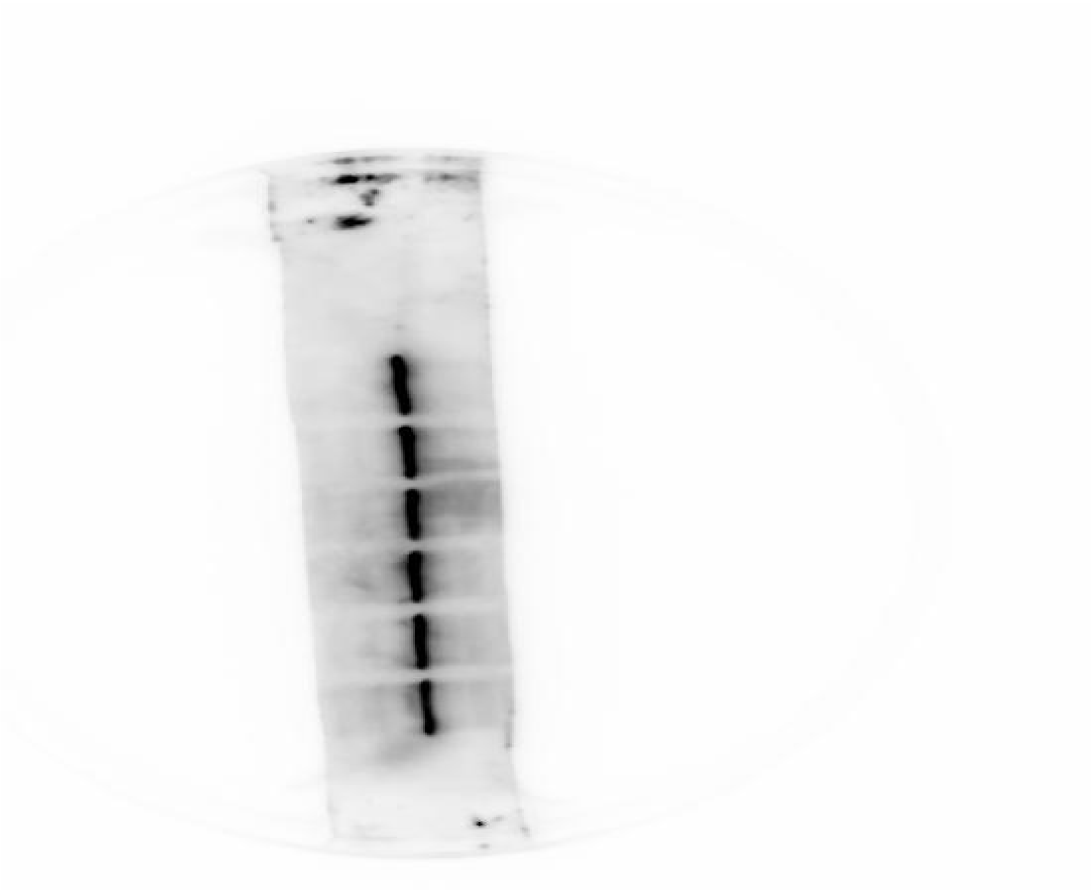

Figure S5C-SIRT1

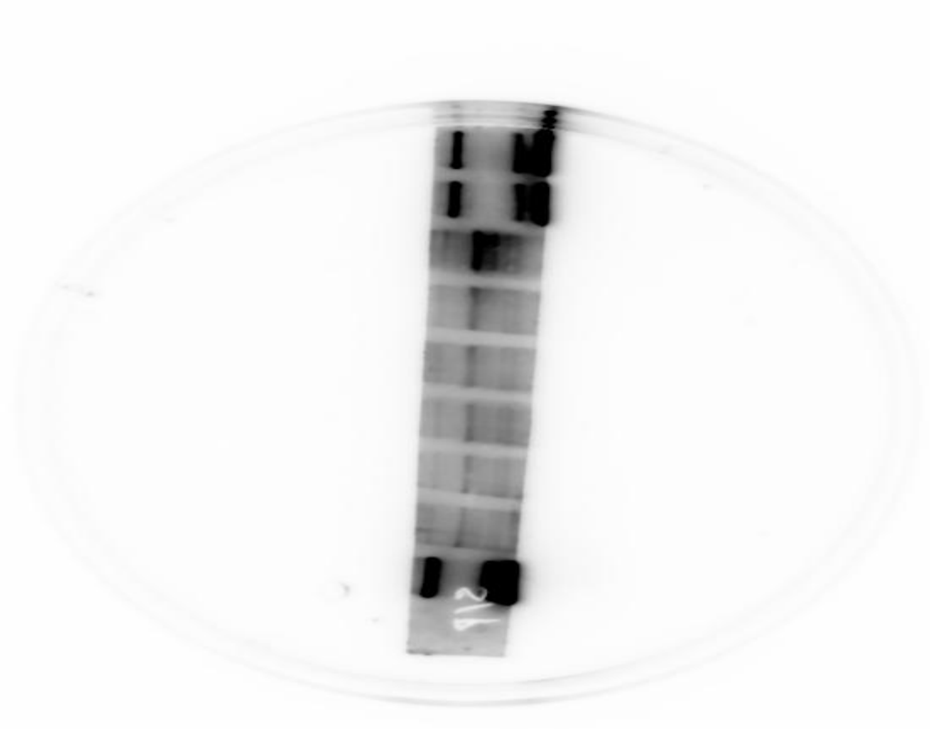

Supplement: Data S1. Original data of Western blot, related to WB analysis and IP section in STAR methods [file mmc1.pdf]
