# Supplementary material for: Ginsenoside Rb1 regulates CPT1A deacetylation to inhibit intramuscular fat infiltration after rotator cuff tear
Source: iScience. 2024 Jun 20;27(7):110331. doi: 10.1016/j.isci.2024.110331 (PMC11277379; doi:10.1016/j.isci.2024.110331)
Supplement: Document S1. Figures S1–S10 [file mmc2.pdf]

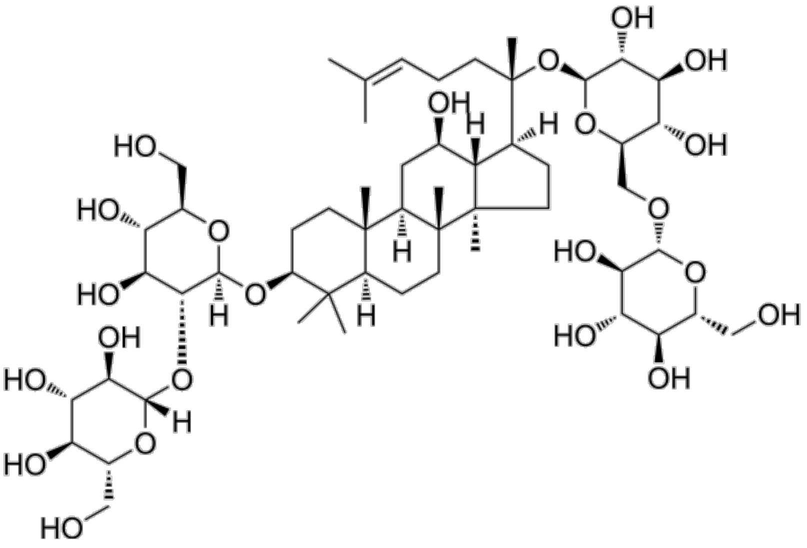

Supplementary Figure 1. the chemical structure of Ginsenoside Rb1, related to figure 1

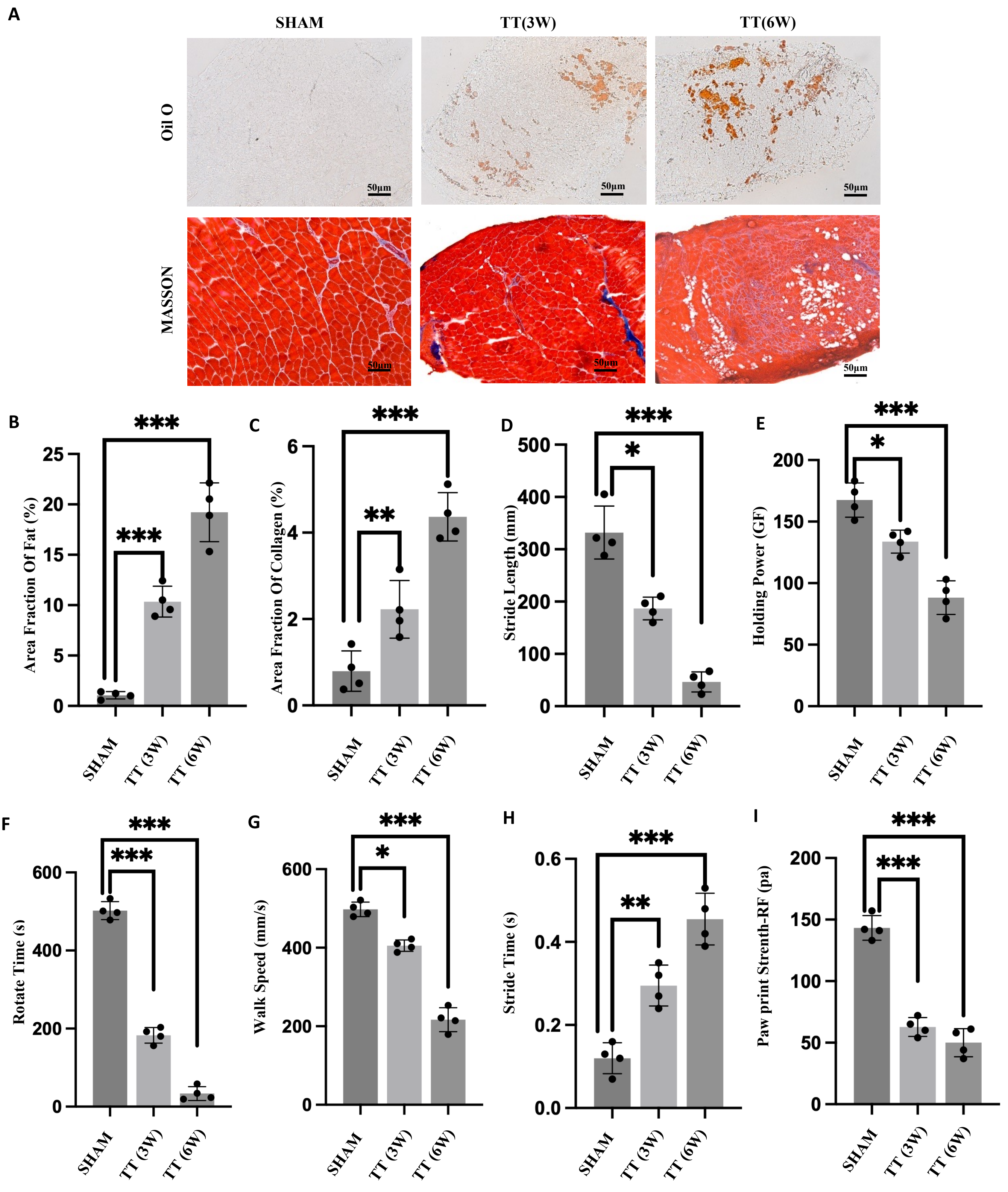

Supplementary Figure 2. Fat infiltration and fibrosis in SS muscle of TT model mice, related to figure 1

The TT mice model was constructed to simulate intramuscular fat infiltration after injury. (A) Oil Red O and Masson Trichrome staining of SS muscle in TT mouse. Scale bars: 50μm. (B) Fat area fraction analysis of Oil Red O staining. (C) Collagen area fraction analysis of Masson Trichrome staining. (D) Measurement of stride length. (E) Measurement of holding power. (F) Measurement of time in rotarod test. (G) Measurement of walk speed. (H) Measurement of stride time. (I) Measurement of paw print strength of right foot. *P<0.05, **P<0.01, ***P<0.001

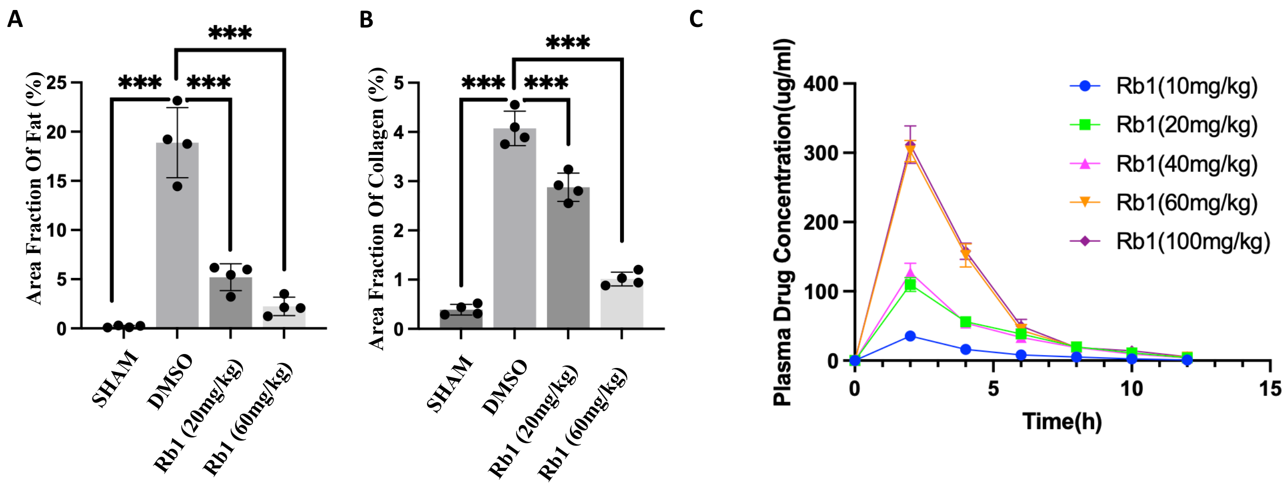

Supplementary Figure 3. Rb1 inhibits fat infiltration of SS muscle while improving shoulder joint function in TT modeling mice, related to figure 2

(A) Fat area fraction analysis of Oil Red O staining. (B) Collagen area fraction analysis of Masson Trichrome staining. (C)Plasma drug concentration of C57 mice treated with ginsenoside Rb1

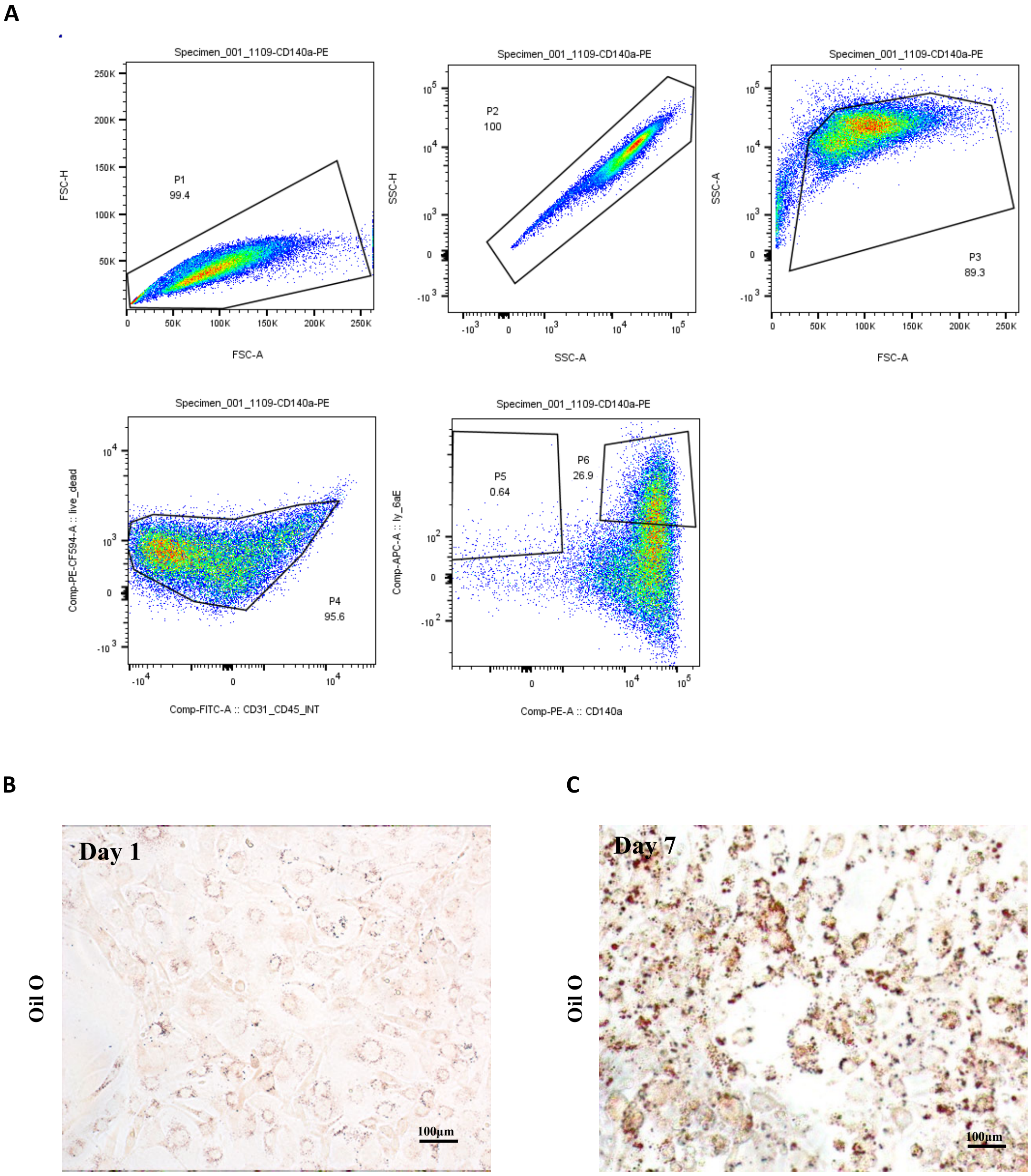

Supplementary Figure 4. Sorting and breeding of FAPs, related to figure 3

(A) Prospective isolation of progenitor populations from skeletal muscle. Expression of CD31-/CD45-/ITGA7-/Sca1+/PDGFRα + population cells were sorted and characterized. (B, C) Sorted cell was cultured with adipogenesis culture medium. Triglycerides were detected by Oil Red O staining after 1 day or 7 days culture.

A B

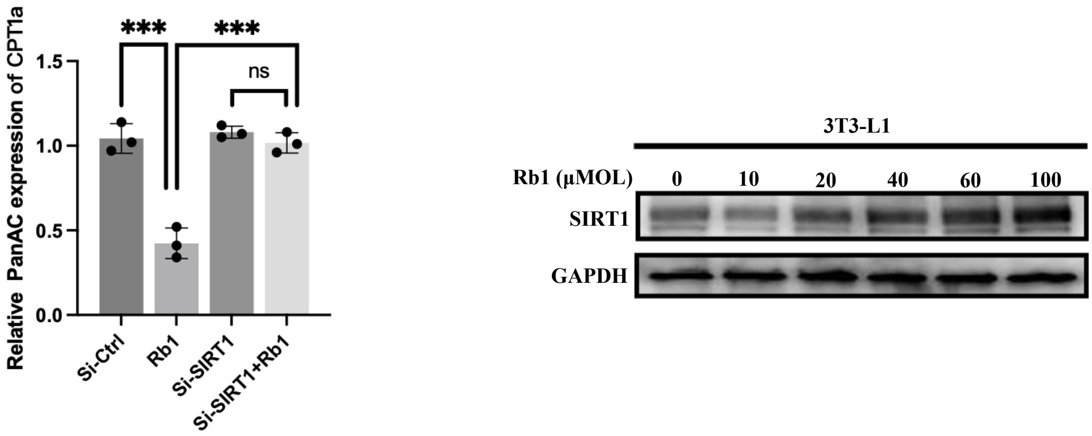

Supplementary Figure 5. SIRT1 is the key enzyme for Rb1 to deacetylate CPT1a,related to figure 5

(A) Relative PanAC protein expression of CPT1a in 3T3-L1 treated with Rb1 or Rb1 combined with Si-SIRT1; (B)Expression of SIRT1 in 3T3-L1 treated with different concentration Rb1 (0,10,20,40,60,100μM) detected by Western Blot. ***$P<0.001$

Pocket1

S	rmsd_refine	E_conf	E_place	E_score1	E_refine	E_score2
-9.9555	3.2433	334.5674	-41.6218	-3.2852	-67.4951	-9.9555
-9.7213	2.8679	343.2141	-68.7742	-11.7407	-59.9800	-9.7213
-9.4712	2.3739	339.8137	-70.0611	-10.0630	-67.2589	-9.4712
-9.3266	2.6867	330.5108	-64.7037	-4.1990	-62.8056	-9.3266
-8.9918	2.7930	339.8808	-78.5401	-9.9470	-58.5201	-8.9918

NAD_Site

S	rmsd_refine	E_conf	E_place	E_score1	E_refine	E_score2
-12.2671	3.3992	408.9990	233.1107	12.5045	-46.5332	-12.2671
-10.1286	2.4253	513.1479	73.7230	13.5565	4.2556	-10.1286
-9.7805	2.0409	512.2920	59.0895	3.3812	9.3054	-9.7805
-9.4780	2.6038	498.4805	-25.8894	-1.2566	12.4734	-9.4780
-9.2303	4.4169	397.7109	59.9865	1.8943	-41.3406	-9.2303

STAC_Site

S	rmsd_refine	E_conf	E_place	E_score1	E_refine	E_score2
-7.3166	3.0228	328.0484	-71.4641	-8.4476	-45.1880	-7.3166
-7.3088	2.9005	330.4262	-53.1219	-10.6060	-43.6453	-7.3088
-7.2326	2.1758	323.2277	-12.1608	-8.0062	-46.7347	-7.2326
-7.0662	1.8958	334.2399	-29.9787	-8.4881	-40.7400	-7.0662
-7.0559	2.0112	342.4586	-35.5113	-8.4127	-41.6133	-7.0559

Supplementary Figure 6. Affinity scoring values of Rb1 with SIRT1, related to figure 6

Affinity score of Rb1 and SIRT1. *rmsd_refine* represents for changes in RMSD values of compounds during induction of fit (Å), *E_Conf* represents for potential energy of compound conformation in docking mode, *E_Place* represents for conformational energy of molecular docking. The interaction energy score of the final docking mode is expressed in S value.

A B

C D

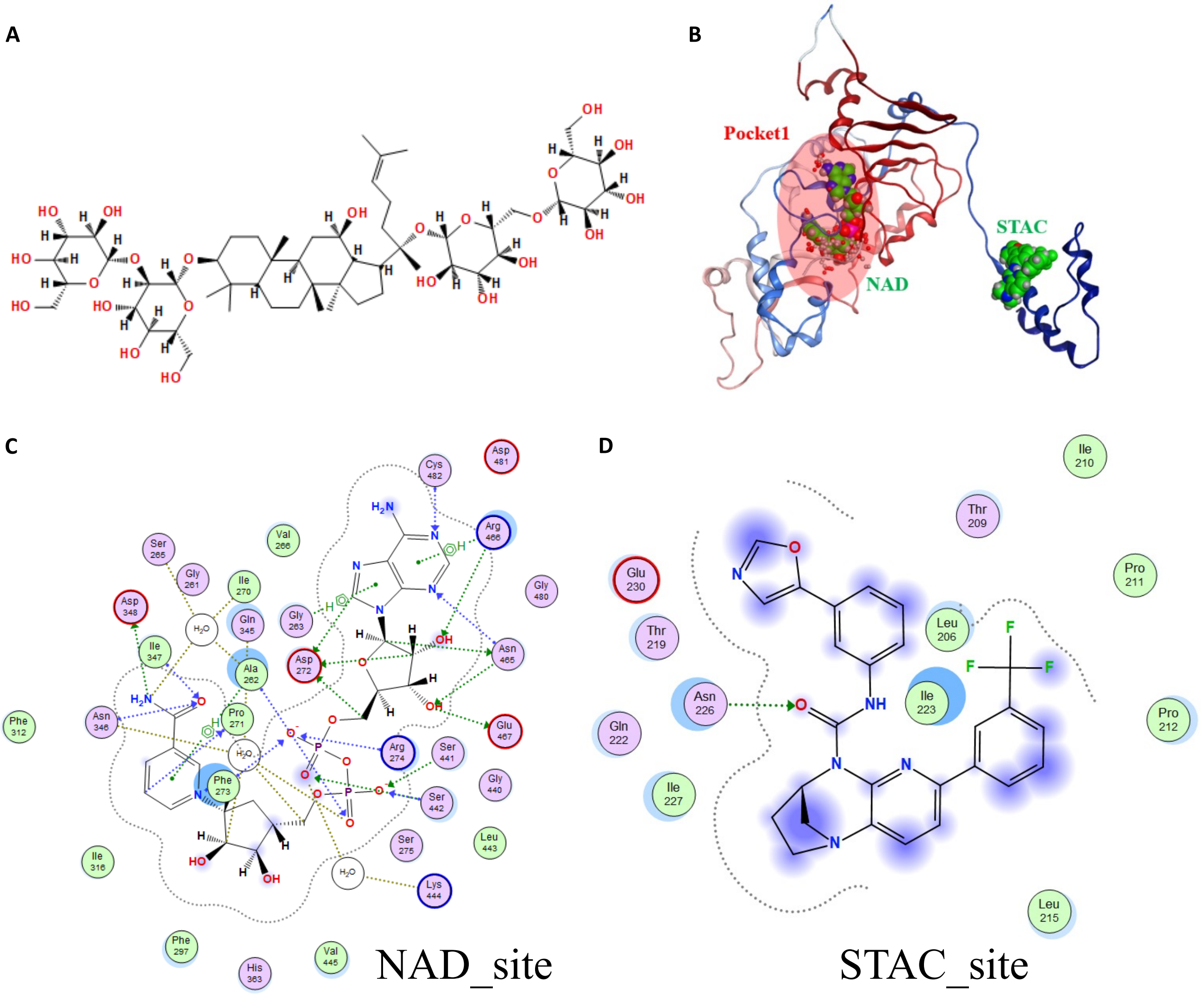

E

Pocket	Size	PLB	Hyd	Side	Residues
1	214	4.53	64	129	1:(**GLY261 ALA262 GLY263 VAL264 SER265 VAL266 SER267 ILE270 PRO271 ASP272 PHE273 ARG274 SER275 ARG276 ASP277 GLY278 ILE279 TYR280** ALA281 PRO293 GLN294 PHE297 PHE312 ILE316 SER324 **GLN345 ASN346 ILE347 ASP348 HIS363** ILE411 VAL412 PHE414 **GLY440 SER441 SER442** LEU443 LYS444 VAL445 **ASN465 ARG466 GLU467** GLY480 ASP481 **CYS482** ASP483 TYR658 SER659 ASP660 SER661 GLU662 ASP663 ASP664)

Supplementary Figure 7. Structure of SIRT1 based on 4ZZJ modeling, related to figure 6

(A) The chemical structural formula of Rb1. (B) The composite structure of human Sirt1 binding agonist is displayed in red and blue streamers, with NAD^+ and STAC in green. (C) 2D Interaction Mode of Sirt1 and NAD^+. (D) 2D Interaction Mode of SIRT1 and STAC. (E) The properties of pocket 1 (size, sparsity Water atomic number, pocket mass score, information including amino acids, etc.). The active site was marked in red and the NAD^+ binding site was marked in yellow.

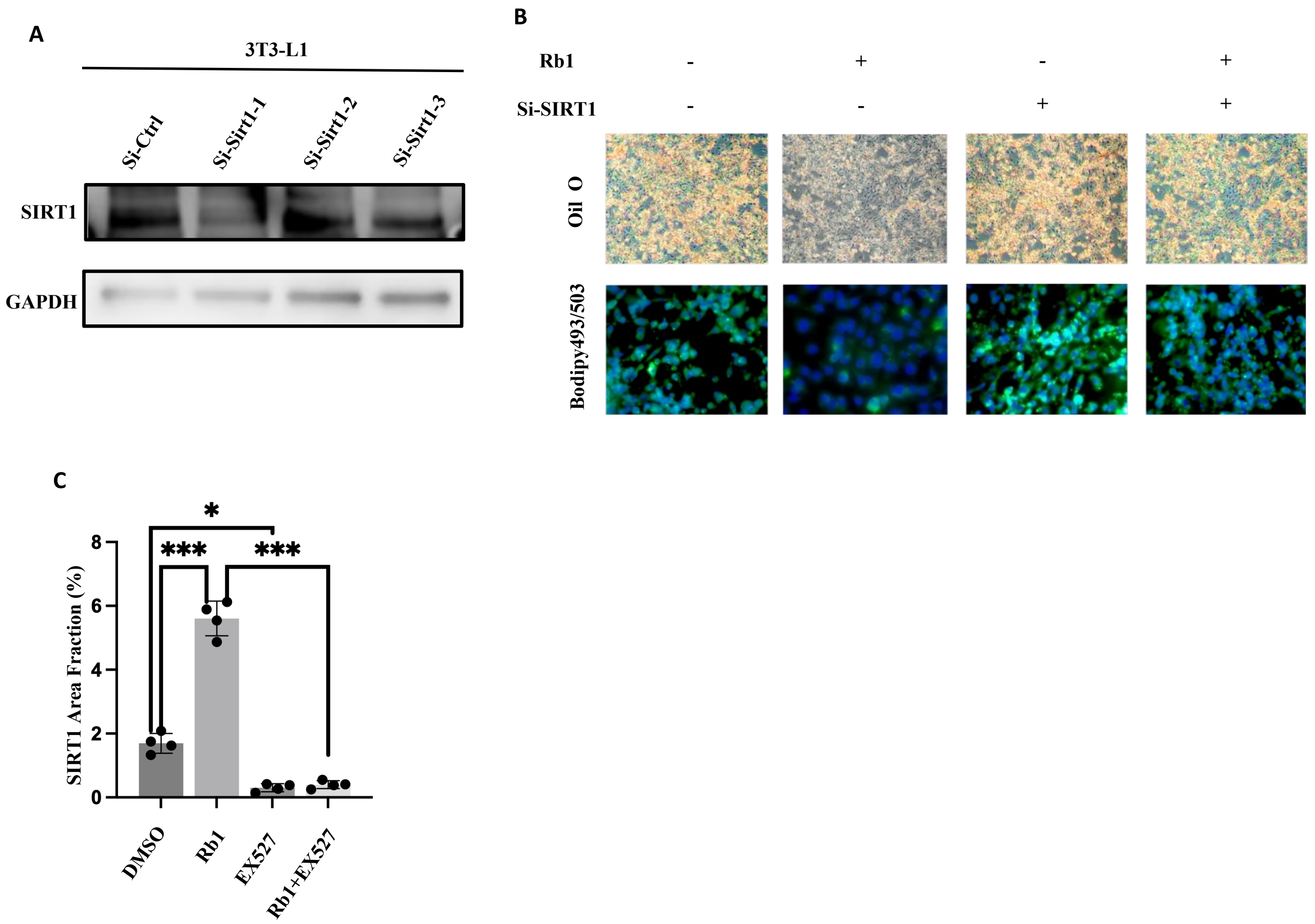

Supplementary Figure 8. Rb1 regulates the CPT1A deacetylation via SIRT1, related to figure 6

(A) Expression of SIRT1 in 3T3-L1 transfected with si-SIRT1 detected by western blot. (B) Oil Red O and Bodipy 493/503 staining of FAPs treated with Rb1 and Si-SIRT1. (C) SIRT1 area fraction= area of SIRT1(red)/entire sample area. *P<0.05, ***P<0.001

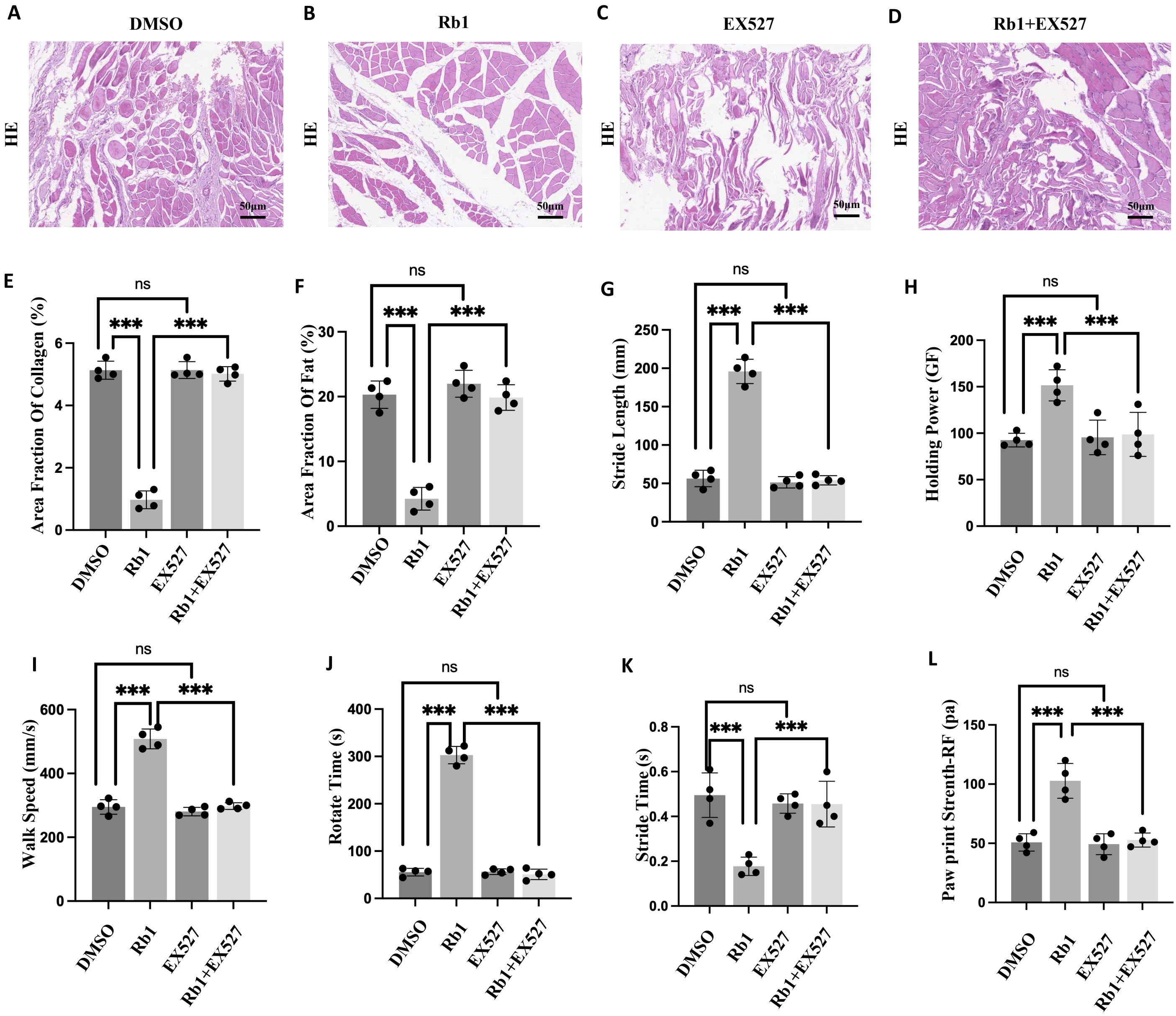

Supplementary Figure 9. Rb1 regulates the CPT1A deacetylation via SIRT1 in vivo, related to figure 6

(A-D) HE staining of SS muscle in TT mouse treated with Rb1 and EX527 separately and together.(n=4 for each group) Scale bars: 50μm. (E) Collagen area fraction analysis of Masson Trichrome staining of SS muscle in TT mouse treated with Rb1 and EX527 separately and together. (F) Fat area fraction analysis of Oil Red O staining. (G-L) measurement of stride length, holding power, walk speed, time in rotary test, stride time and paw print strength of right foot of TT mouse treated with Rb1 and EX527 separately and together. ****P*<0.001

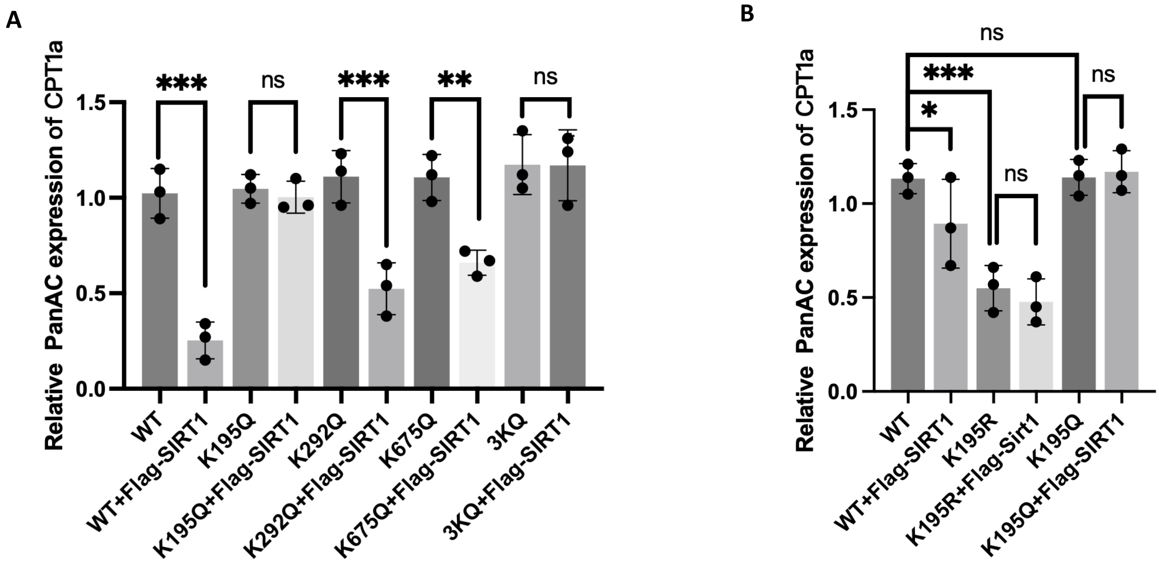

Supplementary Figure 10. Rb1 regulates lipolysis via affecting the CPT1A deacetylation at K195 site, related to figure 7

(A) Relative Acetylation level of CPT1A in 3T3-L1 cells transfected with Flag-SIRT1 and wild type CPT1A (WT) or the different lysine-to-arginine mutants (K195Q, K292Q, K675Q, 3KQ) together; (B) Relative Acetylation level of CPT1A in 3T3-L1 cells transfected with Flag-SIRT1 and CPT1A WT or the k195Q or 195 lysine-to-glutamine (k195R) mutants together. ****P*<0.001
